# Magnolol Prevents Acute Alcoholic Liver Damage by Activating PI3K/Nrf2/PPARγ and Inhibiting NLRP3 Signaling Pathway

**DOI:** 10.3389/fphar.2019.01459

**Published:** 2019-12-05

**Authors:** Xiao Liu, Yanan Wang, Di Wu, Shuangqiu Li, Chaoqun Wang, Zhen Han, Jingjing Wang, Kai Wang, Zhengtao Yang, Zhengkai Wei

**Affiliations:** ^1^College of Life Sciences and Engineering, Foshan University, Foshan, China; ^2^College of Veterinary Medicine, Jilin University, Changchun, China

**Keywords:** alcoholic liver disease, magnolol, oxidative stress, NLRP3, PPARγ

## Abstract

Alcoholic liver damage (ALD) is a toxic liver damage caused by excessive drinking. Oxidative stress is one of the most crucial pathogenic factors leading to ALD. Magnolol is one of the main active constituents of traditional Chinese medicine *Magnolia officinalis*, which has been reported to possess many pharmacological effects including anti-inflammatory, anti-oxidant, and anti-tumor. However, the effects of magnolol on ALD remain unclear. In this study, we firstly evaluated the protective effects of magnolol on ALD, and then tried to clarify the mechanism underlying the pharmacological activities. AST, ALT, GSH-Px, and SOD were detected by respective kits. Histopathological changes of liver tissue were analyzed by H&E staining. The activities of PI3K, Nrf2, and NLRP3 signaling pathways activation were detected by western blotting analysis. It was showed that alcohol-induced ALT and AST levels were significantly reduced by magnolol, but the antioxidant enzymes of GSH-Px and SOD levels were significantly increased. Magnolol attenuated alcohol-induced pathologic damage such as decreasing hepatic cord swelling, hepatocyte necrosis, and inflammatory cell infiltration. Furthermore, it was found that magnolol inhibited oxidative stress through up-regulating the activities of HO-1, Nrf2, and PPARγ and the phosphorylation of PI3K and AKT. And magnolol also decreased inflammatory response by inhibiting the activation of NLRP3inflammasome, caspase-1, and caspase-3 signaling pathway. Above results showed that magnolol could prevent alcoholic liver damage, and the underlying mechanism was through activating PI3K/Nrf2/PPARγ signaling pathways as well as inhibiting NLRP3 inflammasome, which also suggested magnolol might be used as a potential drug for ALD.

## Introduction

Alcoholic liver damage caused by excessive drinking accounts for an important proportion in liver diseases and the incidence and mortality of ALD have been increased in recent years (Koneru et al., 2017; Tang, et al., 2017). The course of ALD usually manifests as fatty liver at the beginning and turns into alcoholic hepatitis, alcoholic liver fibrosis, and alcoholic cirrhosis. Excessive drinking can also induce extensive hepatocyte necrosis or even liver failure during severe alcohol abuse (Banerjee et al., 2013; Liu et al., 2017).

Liver is the most important organ that metabolizes alcohol in the body, the ingested alcohol enters the blood circulation after being absorbed in the digestive tract, and 95% of the alcohol is metabolized by the liver (Glade and Meguid, 2017). There are three ways in the metabolism of alcohol in the body: the alcohol dehydrogenase (ADH) system, hepatic microsomal ethanol-oxidizing system (MEOS), and catalase (CAT) system (Sugimoto and Takei, 2017). By these ADH, MEOS, and CAT systems, alcohol is denatured into acetaldehyde (Jeong et al., 2000; Crabb and Liangpunsakul, 2007). Acetaldehyde causes mitochondrial function disorders (Crabb and Liangpunsakul, 2007; Kim et al., 2016). In addition, acetaldehyde can also combine with various proteins to form acetaldehyde addenda, which acts as an antigen to cause an immune reaction, thereby participating in the occurrence and development of liver damage (Morimoto et al., 1995; Niemela et al., 1998).

It has been reported that oxidative stress is recognized as one of the most important pathogenesis of ALD (Liu et al., 2017; Liu et al., 2018;). When a large amount of alcohol is consumed, the acetaldehyde, an intermediate of alcohol metabolism, greatly increases the amount of reactive oxygen species (ROS) in the body, causing oxidative stress in the liver (Kim et al., 2016; Grander et al., 2018). The antioxidants in the body will be consumed in large quantities, and the antioxidant system will not perform its normal function, especially the protection of the liver under these circumstances (Kim et al., 2016; Sugimoto and Takei, 2017).

Magnolol is the main chemical component of the traditional Chinese medicine *Magnolia officinalis* (Chen et al., 2009). It is reported that magnolol can alleviate dextran sulfate sodium-induced colitis through regulating inflammation and mucosal damage in mice (Zhao et al., 2017). Other researchers have reported toxicity tests of magnolol *in vivo* and *in vitro*. Magnolol was concentrated at 240 mg/kg without adverse effects on the body (Sarrica et al., 2018). Magnolol also inhibits the proliferation and invasion of cholangiocarcinoma cells by inhibiting NF-κB signaling pathway (F. H. Zhang et al., 2017) and promotes heat production and attenuates oxidative stress in 3T3-L1 adipocytes (Parray et al., 2018). Although many studies have demonstrated the pharmacological effects of magnolol on above diseases, the effect of magnolol in preventing ALD through antioxidant and anti-inflammatory pathways has not been reported. Thus, in this study, we aimed to investigate the effects of magnolol on ALD and explore its underlying mechanisms.

## Materials and Methods

### Chemicals and Reagents

Magnolol was purchased from National Institutes for Food and Drug Control (Beijing, China). Ethanol was bought from Beijing Chemical Works (Beijing, China). AST, ALT, SOD, iNOS, and GSH-Px kit were provided by Nanjing Jiancheng Bio-engineering Institute (Nanjing, China). Cox-2 and CYP2E1 Elisa kits were bought from Shanghai Lanpai Biotechnology co. LTD. Antibodies against GAPDH, p-AKT, AKT, Nrf2, HO-1, NLRP3, p-PI3K, and PI3K were purchased from Boster bioengineering co. LTD (Wuhan, China). Antibodies against PPARγ and Caspase-3 were obtained from Cell Signal Technology (Boston, MA, USA). Anti-Caspase-1 antibody was bought from Abcam (Cambridge, MA, USA). Additionally, all other chemicals were provided by Beijing Chemical Works (Beijing, China), if not otherwise indicated.

### Animals

Male BALB/c mice (6–8 weeks, 18–22 g) were purchased from Liaoning Changsheng Biotechnology Co., Ltd (Certificate SCXK2010–0001; Liaoning, China). Mice were housed under 12-h light and 12-h dark-protected cycling conditions with the temperature at 24 ± 1°Cand the relative humidity is 50% ± 10%. Adaptive feeding for 5 days before the start of the experiment, during which the mice were free to eat and drink. All animal experiments were approved by the Care and Use of Laboratory Animals of the Jilin University and in accordance with the current Animal Protection Laws of China.

### Experimental Design

The mice were randomly divided into five groups (n = 5 per group) as follows:

- Control group: mice were intraperitoneally injected with 300 μl of 0.9% saline per day.
- Ethanol group: mice were intraperitoneally injected with the same volume of 0.9% saline per day and gavaged with ethanol [15 ml/kg BW, absolute ethanol, the acute oral toxicity limits is 22.5 (18.8-27.0) ml/kg (Kimura et al., 1971)] on the last day.
- Ethanol + 5 mg/kg magnolol group: mice were intraperitoneally injected with magnolol (5 mg/kg BW mixed in 300 μl of 0.9% saline) per day and gavaged with ethanol 1 h later on the last day.
- Ethanol + 10 mg/kg magnolol group mice were intraperitoneally injected with magnolol (10 mg/kg BW mixed in 300 μl of 0.9% saline) per day and gavaged with ethanol 1 h later on the last day.
- Ethanol + 20 mg/kg magnolol group: mice were intraperitoneally injected with magnolol (20 mg/kg BW mixed in 300 μl of 0.9% saline) per day and gavaged with ethanol 1 h later on the last day.

The duration of the whole experiment was 3 days and the mice were sacrificed after 9 h of gavage on the last day. Then the serum was separated and the liver tissue was fixed in formaldehyde or prepared for cryopreservation at -80°C for further use.

### Analysis of AST and ALT

The blood sample was placed in a refrigerator overnight at 4°C and centrifuged at 3,000 rpm (10 min, 4°C) the next day, and then we detected alanine aminotransferase (ALT) and aspartate aminotransferase (AST) in serum *via* the kits purchased from Nanjing Jiancheng Bioengineering Institute. All operating steps were carried out in strict accordance with the instructions, and then the absorbance was measured *via* using a microplate reader. The results were calculated and analyzed.

### Histopathological Examination

The liver tissue was fixed in formaldehyde solution for 48 h and then embedded in paraffin. Paraffin sections were dehydrated using ethanol and stained with hematoxylin-eosin and observed under a light microscope for assessment of histopathological damage.

### SOD and GSH-PX Analysis

Frozen liver tissue (0.5 g) was used for SOD and GSH-PX analysis. The blood was removed by rinsing with ice-cold saline and the liver tissue was wiped clean with filter paper. Cut the liver tissue block as soon as possible and pour it into the pre-cooled homogenate medium (4.5 ml, pH 7.2–7.4) for grind in homogenization. Then the resulting suspension homogenate was centrifuged at 3,000 rpm (10 min, 4°C). The precipitate was discarded and the supernatant was retained for detection. All operations were performed according to the instructions. All the liquids required were mixed and allowed to stand at room temperature for 15 min, and then the absorbance at 412 or 550 nm was measured to determine the level of glutathione peroxidase (GSH-Px) or superoxide dismutase (SOD) of each group.

### qReal-time PCR Analysis

Frozen liver tissue (0.1 g) in a mortar was grinded into powder with liquid nitrogen, and then transferred it to a 1.5 ml centrifuge tube. Total RNA was extracted from liver tissues using TRizol and the RNA samples were dissolved in 0.1% DEPC (diethyl pyrocarbonate) water. RNA samples were reverse transcribed to cDNA using the Maxima H Minus First Strand cDNA Synthesis Kit (Thermo Scientific ™) according to the instructions provided by the supplier. The FS Universal SYBR Green Master (F. Hoffmann-La Roche Ltd.) was used to establish a 25 μl system to detect changes in IL-1β, TNF-α, iNOS (nitric oxide synthase), CYP2E1 (cytochrome P450 2E1), and Cox-2 (cyclooxygenase-2) mRNA expression in liver tissues. The primers used were purchased from Sangon Biotech (Shanghai) Co., Ltd. And qReal-time PCR (qRT-PCR) was performed on a 7500 real-time PCR system (Applied Biosystems, Carlsbad, CA, USA).

### Enzyme Activity Test

Cox-2 and CYP2E1 activities were detected by respective ELISA kits, and iNOS activity was detected by nitric oxide synthase (NOS) typing kit. Frozen liver tissue (0.1 g) was added to 900 μl of physiological saline and centrifuged at 15,000 rpm for 10 min (4°C) to obtain a supernatant. All operations were strictly implemented according to the instructions. The optical density (O.D) of Cox-2 and CYP2E1 was detected at 450 nm using a microtiter plate reader within 15 min, and the absorbance of samples to detect the activity of iNOS was measured at 530 nm using an ultraviolet spectrophotometer.

### Western Blotting Analysis

RIPA (Radio Immunoprecipitation Assay) Lysis and Extraction Buffer (Thermo Scientific ™) was added to frozen liver tissue samples, and they were grinded in a homogenizer. The obtained tissue homogenate was centrifuged at 15,000 rpm (10 min, 4°C) and the supernatant was left. Then the Pierce ™ BCA (bicinchoninic acid) Protein Assay Kit (Thermo Scientific ™) was used to determine protein concentration. Equal amounts of proteins were separated by 10–12% SDS polyacrylamide gels and transferred to polyvinylidene fluoride (PVDF) membranes. The PVDF membranes were blocked with 5% skim milk for 3 h on a shaker at room temperature and then incubated with the primary antibodies (diluted with 5% skim milk) overnight at 4°C, and the concentrations of the primary antibodies are as follows: the concentration of primary antibody used for GAPDH, p-AKT, AKT, Nrf2, HO-1, NLRP3, p-PI3K, PI3K, and Caspase-1 was 0.5 μg/ml, and the concentration of primary antibody used for PPARγ, and Caspase-3 was 1 μg/ml. The membranes were washed *via* Tris-Buffered Saline Tween-20 (TBST) three times for 10 min each time and then incubated with the secondary antibodies (diluted with TBST) on the second day. Finally, the membranes were visualized with the chemiluminescent HRP substrate after washed again by TBST and analyzed *via* Image J gel analysis software.

### Statistical Analysis

All the above data were expressed by means ± SD, and each group of data was analyzed by one-way ANOVA combined with Tukey’s multiple comparison tests for comparison using the GraphPad Prism 6.0 software. Statistical significance was considered as P < 0.05.

## Results

### Magnolol Decreased the ALT and AST Levels in the Serum of Alcohol-Induced Liver Damage

**Figure 1:**
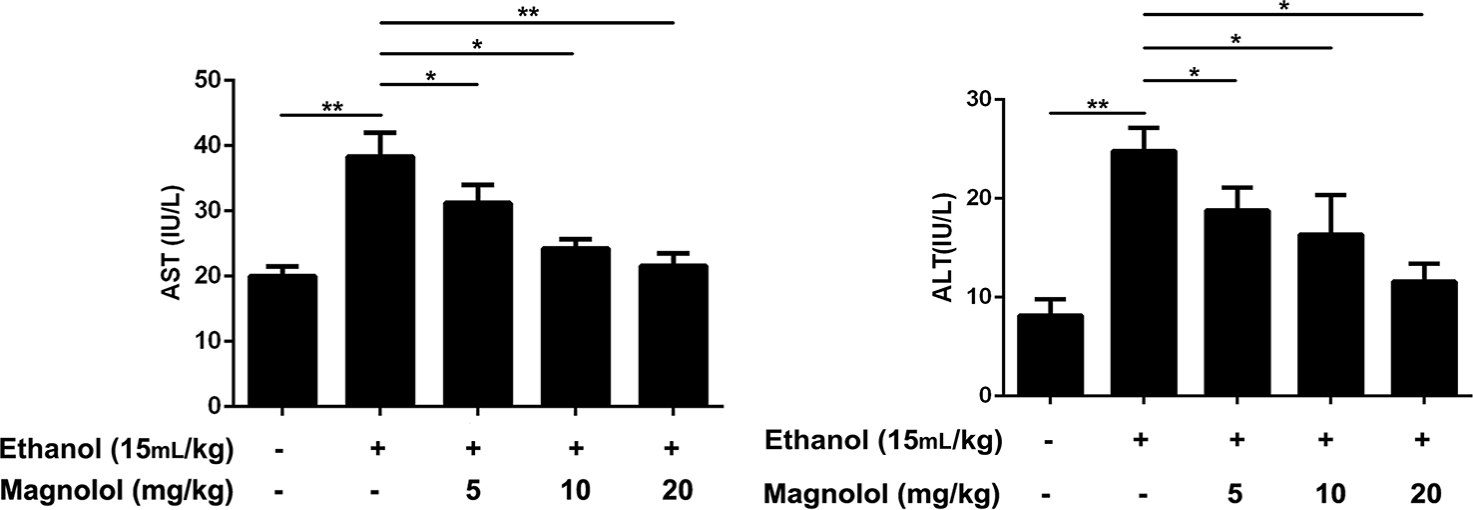
Effects of magnolol on mice alcohol-induced liver damage in ALT and AST levels. Magnolol was given for 3 days and alcohol was gavaged 10 h after the last dose. We used the blood to detect the serum AST and ALT. The data were demonstrated as means ± SD. (*P < 0.05, **P < 0.01).

AST and ALT are biomarkers for liver damage. AST and ALT levels in serum were examined by respective kits. As showed in **Figure 1**, the AST and ALT levels in the alcohol group were significantly higher than control group. In contrast, the levels of AST and ALT in the magnolol treated groups were significantly reduced, especially in the highest concentration group (20 mg/kg), which was close to the control group.

**Figure 2:**
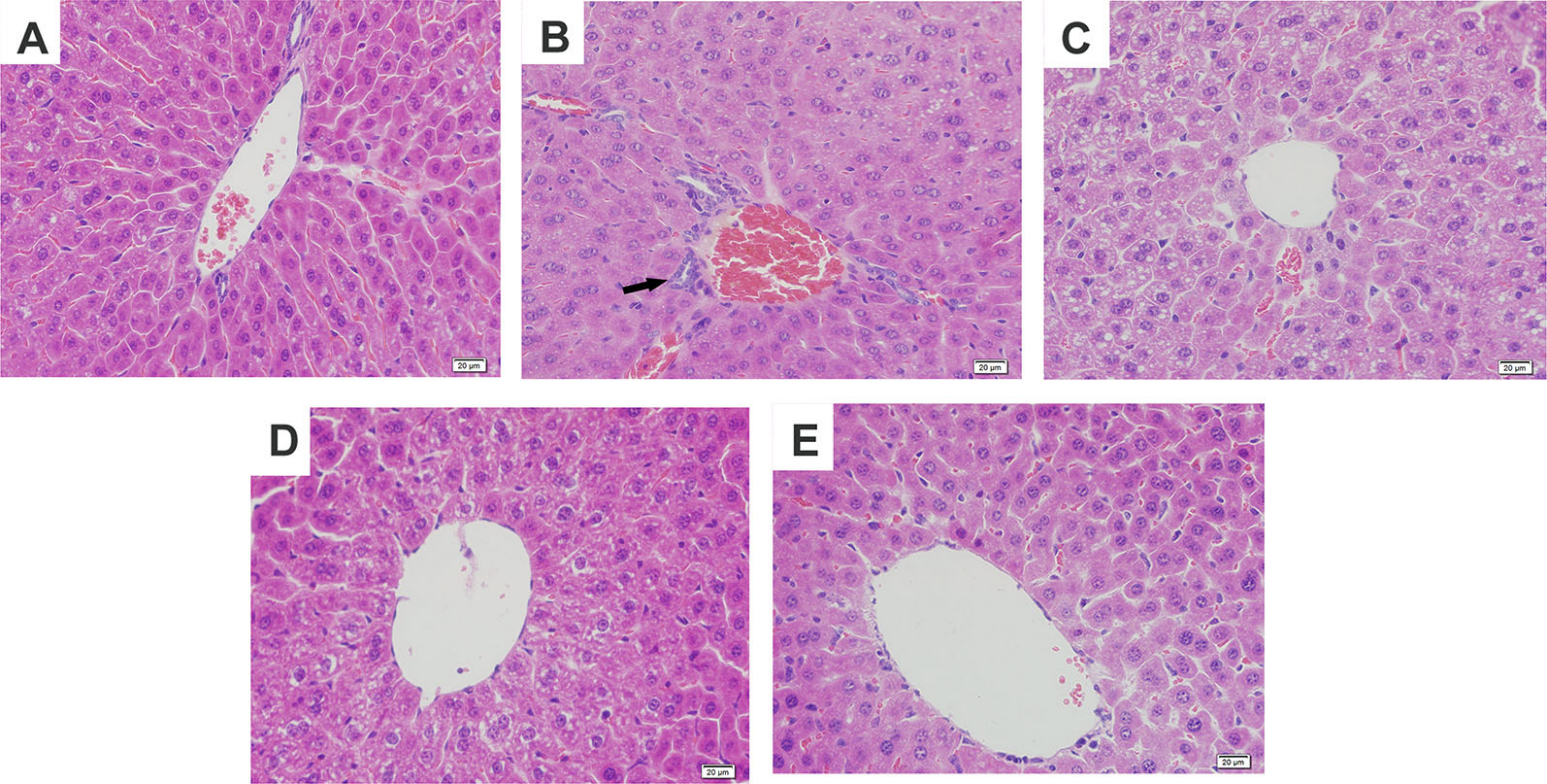
Effects of magnolol on histopathological changes of liver tissues in alcohol-induced liver damage. **(A)** Control. **(B)** Alcohol. **(C)** Alcohol + magnolol (5 mg/kg). **(D)** Alcohol + magnolol (10 mg/kg). **(E)** Alcohol + magnolol (20 mg/kg). The black arrow in **(B)** represents inflammatory cell infiltration.

### Magnolol Attenuated Alcohol-Induced Liver Damage in Mice

To further confirm the protective effects of magnolol on alcohol-induced liver damage, histopathological examination was carried out. It was found in **Figure 2** that alcohol caused hepatic cord swelling, hepatocyte necrosis, and inflammatory cell infiltration, but magnolol significantly improved the above pathological conditions.

### Magnolol Increased the Antioxidant Enzymes of SOD and GSH-Px in ALD

**Figure 3:**
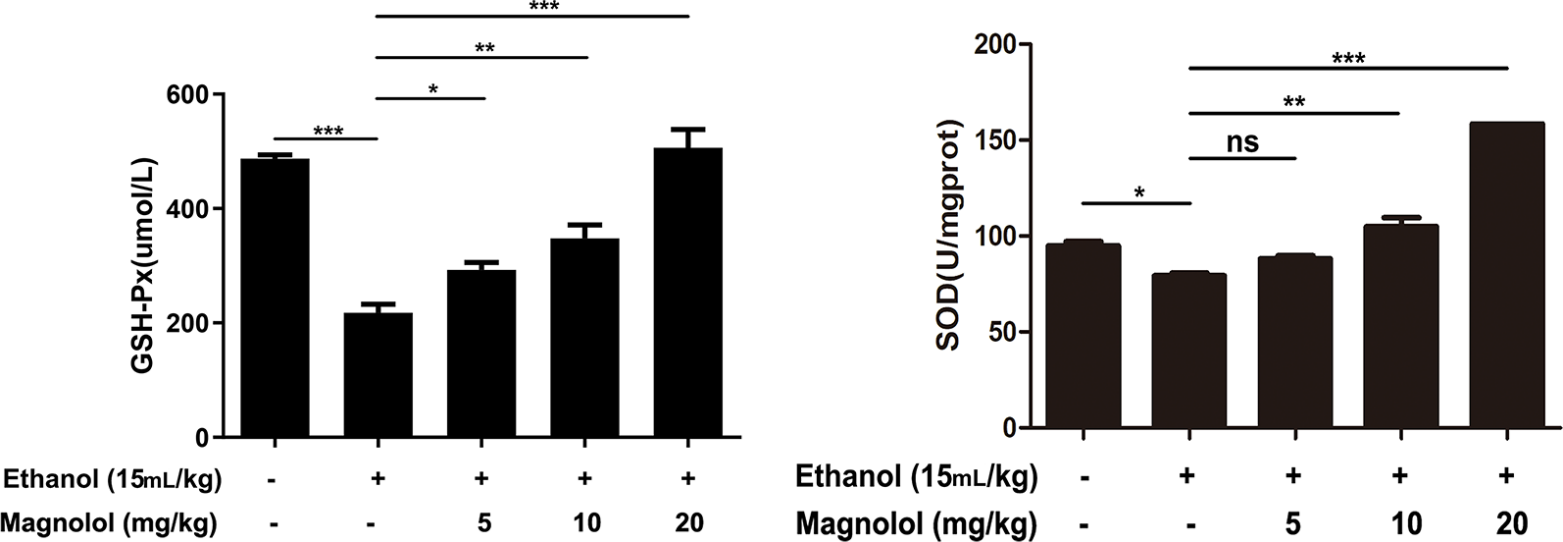
Effects of magnolol on mice alcohol-induced liver damage in SOD and GSH-Px level. Before alcohol was gavaged, magnolol was given for 3 days. We took the liver tissue to measure SOD and GSH-Px. The date is presented as mean ± SD. (*P < 0.05, **P < 0.01, ***P < 0.001and "ns" means not significant).

SOD and GSH-Px are antioxidant enzymes that represent the capacity of liver. To further investigate the mechanism by which magnolol prevented alcoholic liver damage, we examined the levels of SOD and GSH-Px in liver. The results showed that the levels of SOD and GSH-Px were significantly decreased in the alcohol-treated mouse liver. However, magnolol pretreatment reversed this result, and the values of SOD and GSH-Px gradually increased in a dose-dependent manner (**Figure 3**).

### Magnolol Down-Regulated the Expression of iNOS, Cox-2, and CYP2E1 in Alcoholic Liver Damage

**Figure 4:**
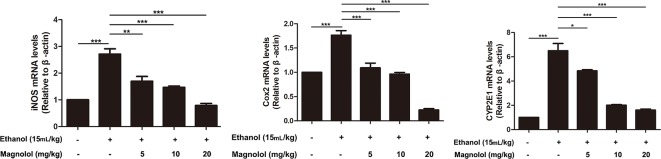
Effects of magnolol on mice alcohol-induced liver damage in iNOS, Cox-2, and CYP2E1 at the genetic level. q-RT PCR analysis was used to detect the expression of iNOS, Cox2, and CYP2E1 at the genetic level. The data is presented as mean ± SD. (*P < 0.05, **P < 0.01, ***P < 0.001).

**Figure 5:**
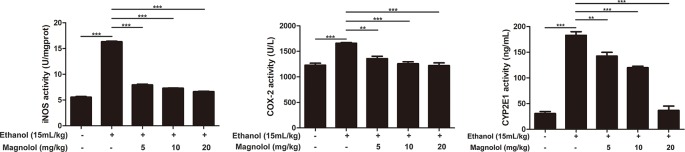
Effects of magnolol on mice alcohol-induced liver damage in iNOS, Cox-2, and CYP2E1 at the enzyme level. iNOS, Cox-2, and CYP2E1 were examined by enzyme activity test. The data is presented as mean ± SD. (**P < 0.01, ***P < 0.001).

iNOS and Cox-2 are usually activated in the process of oxidative stress and inflammation, which result in tissue damage (Song et al., 2018). CYP2E1 is also a key enzyme in regulating alcoholic liver damage. The effect of magnolol on the expressions and the enzymatic activities of ALD, iNOS, Cox-2, and CYP2E1 was investigated in qRT-PCR and ELISA methods. The results showed that alcohol significantly increased iNOS, Cox-2, and CYP2E1 in the liver, but magnolol pretreatment markedly down-regulated the expression of these enzymes at the level of gene and enzyme expression (**Figures 4** and **5**).

### Magnolol Upregulated the AKT/PI3K Signaling Pathway in ALD

**Figure 6:**
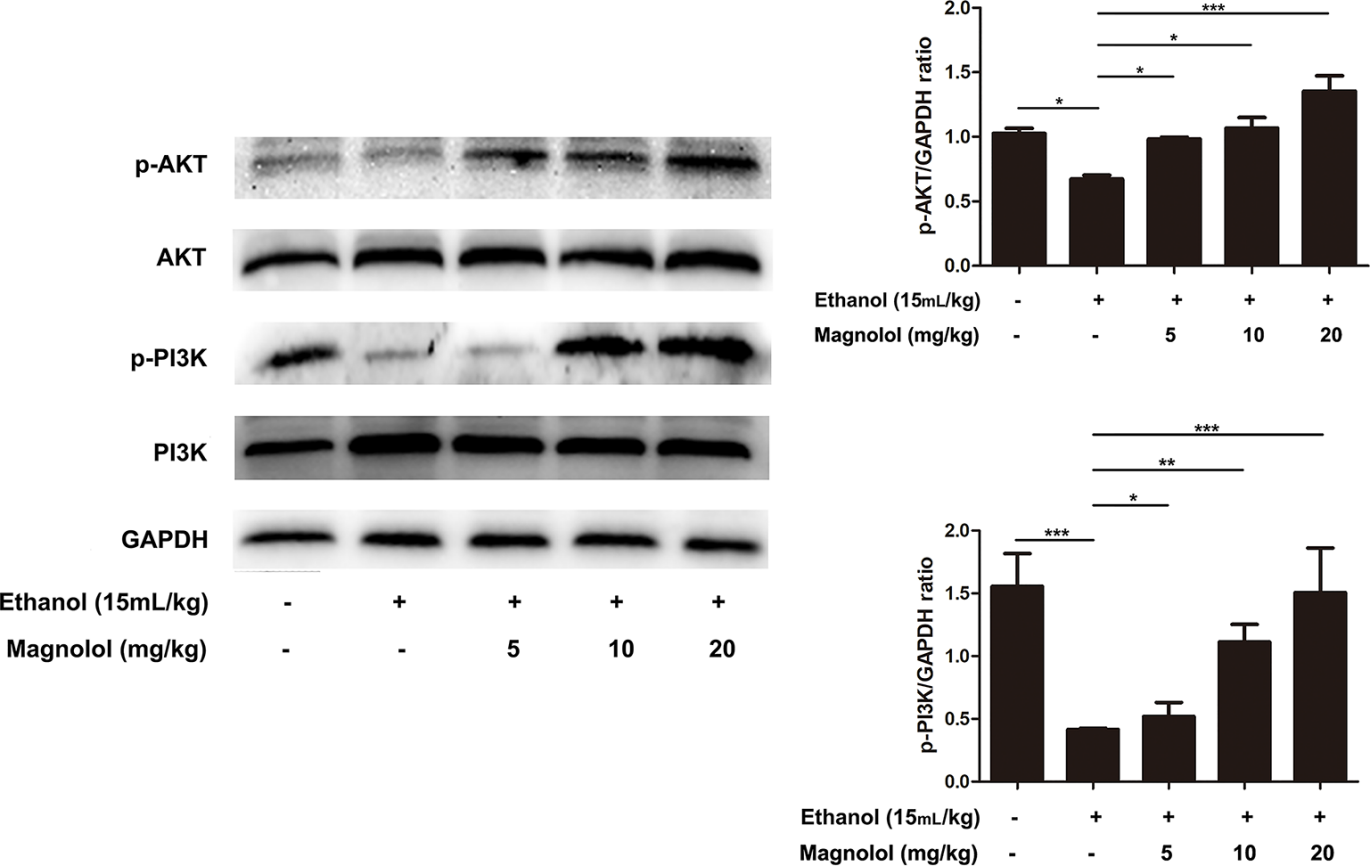
Effects of magnolol on mice alcohol-induced liver damage in the AKT/PI3K signaling pathway. Liver tissues were extracted for protein analysis by western blotting. AKT and PI3K, proteins expression were detected. The levels of AKT and PI3K were compared with GAPDH. The data were demonstrated as means ± SD. (*P < 0.05, **P < 0.01, ***P < 0.001).

The association of magnolol in alcoholic liver damage through the AKT and PI3K signaling pathways was analyzed through measuring the expression levels of AKT and PI3K within Western blotting. As shown in **Figure 6**, alcohol decreased the phosphorylation of AKT and PI3K, but these trends were significantly changed by magnolol in a dose-dependent manner. This result indicated that magnolol could weaken the damage caused by inhibiting oxidative stress and play a critical protective effect in the process of alcoholic liver damage.

### Magnolol Pretreatment Activated Alcohol-Inhibited Nrf2/HO-1 Signaling Pathway

**Figure 7:**
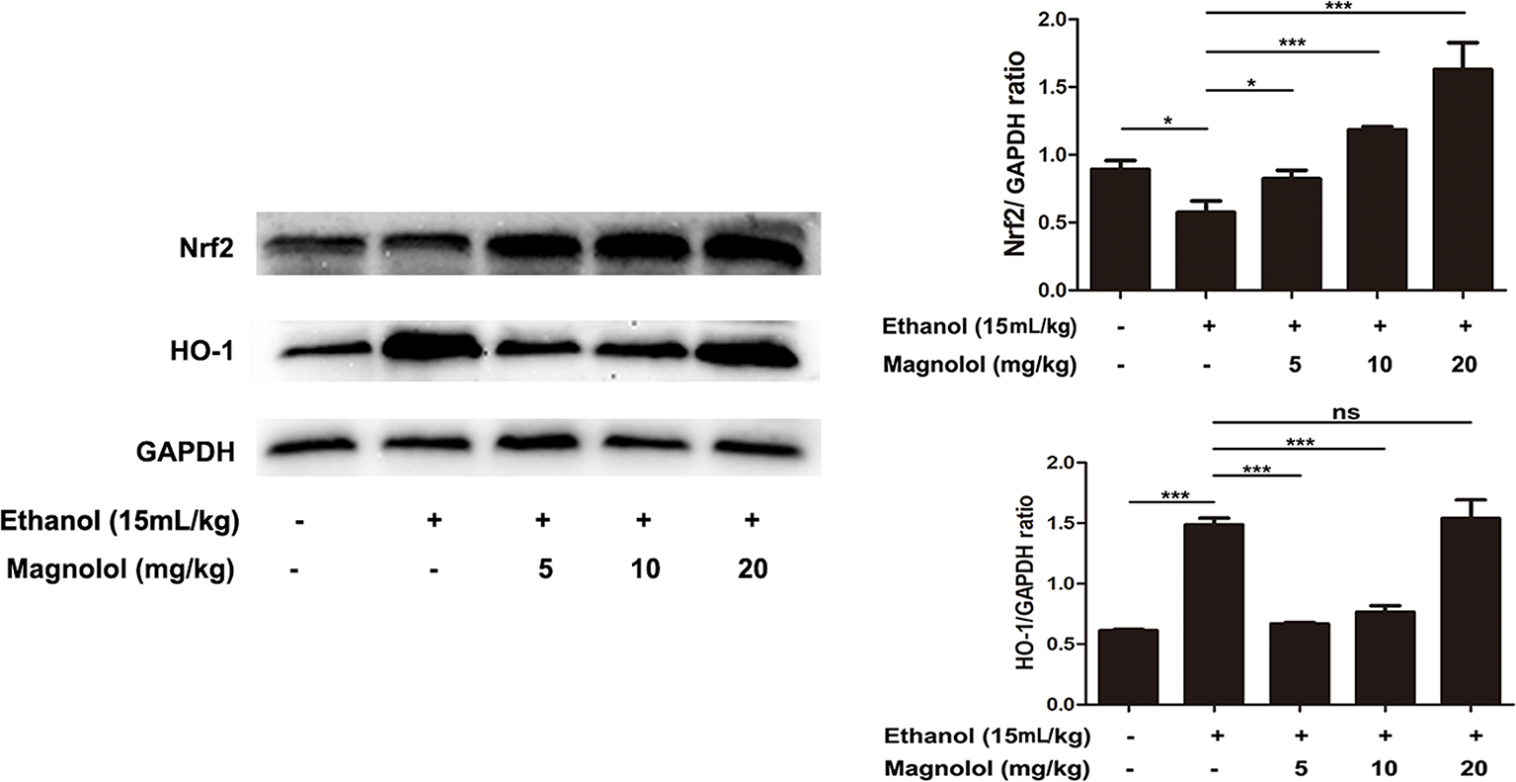
Effects of magnolol on mice alcohol-induced liver damage in the Nrf2/HO-1 signaling pathway. After the completion of modeling and samples were collected, the liver of mice was lysed to detect the proteins by western blotting analysis. The levels of Nrf2 and HO-1 were compared with GAPDH. The data were demonstrated as means ± SD. (*P < 0.05, ***P < 0.001 and "ns" means not significant).

It is reported that Nrf2 is a downstream target of AKT/PI3K signaling pathway (Ali et al., 2018). And Nrf2/HO-1 signaling pathway takes part in oxidation resistance process, whether magnolol also exerts a protective effect on alcoholic liver damage by regulatingNrf2/HO-1signaling pathway has been unknown. So the effect of magnolol on Nrf2/HO-1 signaling pathway in alcoholic liver damage was detected. As shown in **Figure 7**, alcohol reduced the expression of Nrf2, but increased HO-1 expression. It may be due to that HO-1 is an inducible enzyme, acute alcohol stimulation shows an increased reactivity in the early stages. But, once a cascade of uncontrolled outbreaks (such as in alcoholic hepatitis) occurs during stress, it may result in excessive consumption of HO-1 in this process leading to a decrease in its level (Liu et al., 2018). However, magnolol effectively enhanced the activation of Nrf2 and HO-1 in a dose-dependent way. Suggesting that oxidative stress inhibited by magnolol partly owed to activating the Nrf2/HO-1 signaling pathway. Western blotting analysis showed that the protein expression of Nrf2 and HO-1 could be increased to normal level when the concentration of magnolol was 5 mg/kg, which indicated that Nrf2 and HO-1 signaling pathways may be the key targets of magnolol.

### Magnolol Protected the Liver From Alcohol Damage by Activating PPARγ

**Figure 8:**
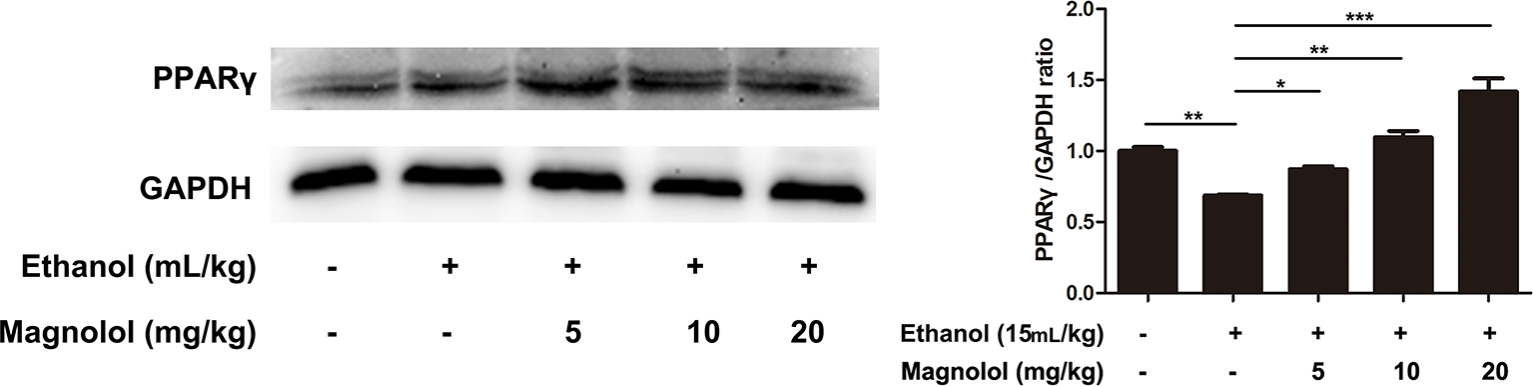
Effects of magnolol on mice alcohol-induced liver damage in PPARγ expression. The collected samples were analyzed for the expression of PPARγ using western blotting analysis. The expression of PPARγ was compared with GAPDH. The data were demonstrated as means ± SD. (*P < 0.05, **P < 0.01, ***P < 0.001 and "ns" means not significant).

It is reported that PPARγ and Nrf2 have a mutually regulated relationship (Lee, 2017). To further explore the relevant mechanisms, the expression of PPARγ was detected by western blotting analysis. As shown in **Figure 8**, alcohol stimulation led to a significant decrease in the expression of PPARγ protein, but the treatment of magnolol reversed this result, and as the concentration of magnolol increased, the expression of PPARγ increased. It was proved that PPARγ was also involved in the antioxidant process of magnolol.

### Magnolol Decreased the Expression of Inflammatory Cytokines IL-1β and TNF-α in Alcoholic Liver Damage

**Figure 9:**
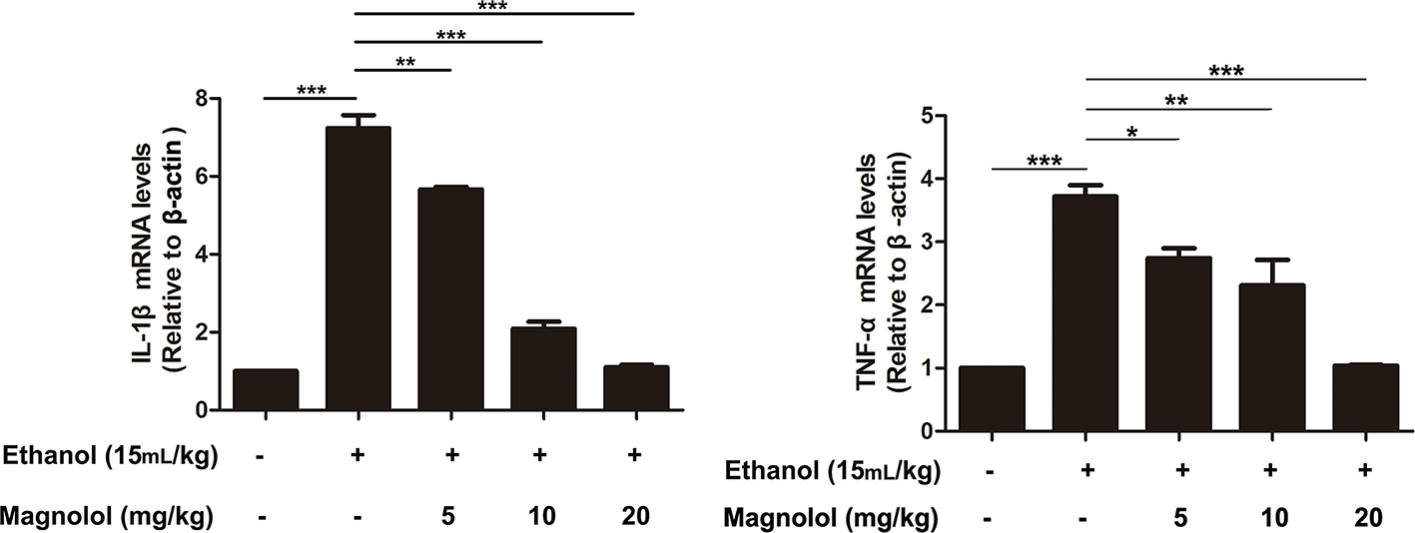
Effects of magnolol on the expression of inflammatory cytokines IL-1β and TNF-α in alcoholic liver damage. Acute alcohol attack stimulated the up-regulation of TNF-α and IL-1β at mRNA measured by q-RT PCR analysis. Magnolol pretreatment significantly inhibited the up-regulation of TNF-α and IL-1β in ALD mice. The data is presented as mean ± SD. (*P < 0.05, **P < 0.01, ***P < 0.001)

ALD usually accompanied with the activation of inflammatory cytokines such as IL-1β and TNF-α (Tang et al., 2015). qRT-PCR analysis was used to detect the effects of magnolol on the expression of inflammatory cytokines in the process of ALD. As presented in **Figure 9**, alcohol enhanced the expression of IL-1β and TNF-α compared with the control group, but magnolol significantly decreased the expression of IL-1β and TNF-α.

### Magnolol Inhibited NLRP3 Inflammasome, Caspase-1, and Caspase-3 Signaling Pathway in ALD Mice

**Figure 10:**
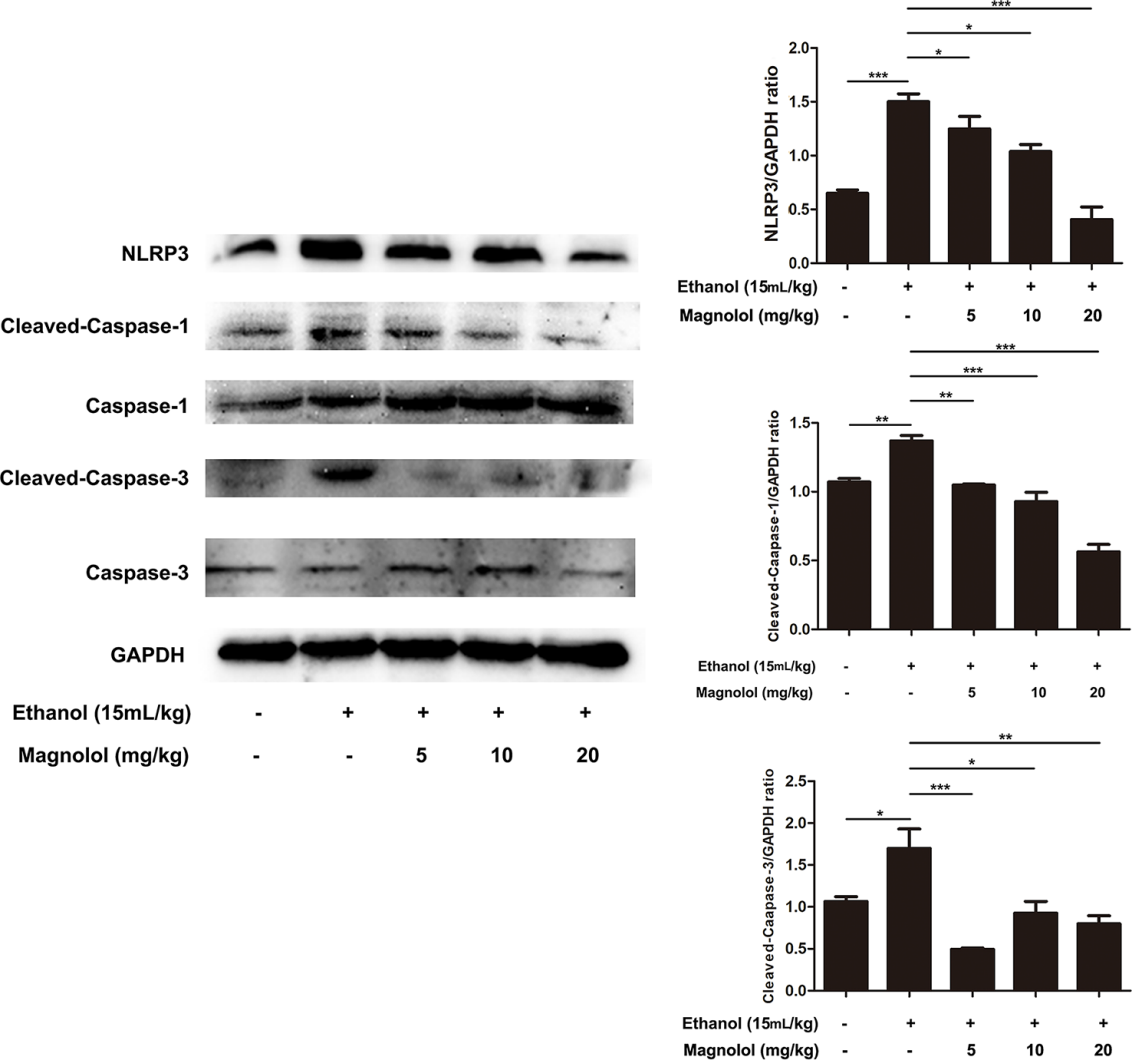
Effects of magnolol on NLRP3 inflammasome, caspase-1 and caspase-3 signaling pathway in ALD mice. Magnolol was given to mice for three times, and then alcohol was gavaged. The level of NLRP3 inflammasome, caspase-1 and caspase-3 was detected by western blotting analysis with the compared with the internal control (GAPDH). The data is presented as mean ± SD. (*P < 0.05, **P < 0.01, ***P < 0.001).

Reports showed that NLRP3 plays an important role in the inflammatory response and the maturation of IL-1β. Western blotting analysis showed that alcohol elevated the activities of NLRP3, Cleaved-Caspase-1, and Cleaved-Caspase-3 (**Figure 10**). However, magnolol pretreatment significantly inhibited alcohol-activated these proteins expressions (**Figure 10**), suggesting that magnolol also prevents inflammatory responses of ALD *via* inhibiting NLRP3 inflammasome, caspase-1, and caspase-3 signaling pathway.

## Discussion

Many studies have reported the pathogenesis of ALD, and more drugs with less toxic and side effect for clinic treatment are urgently needed. Silymarin is currently recognized as an effective treatment for ALD due to its excellent role in ALD treatment and its excellent safety record (Saller et al., 2001). It is reported that silymarin significantly attenuated alcoholic liver injury was through inhibiting NF-κB signaling pathway and reducing excess oxygen free radicals (Song et al., 2006; Zhang et al., 2013). Magnolol is now widely used in clinical treatment like antidepressants and anti-inflammatory. Magnolol abrogates depressive-like behaviors by inhibiting neuroinflammation and oxidative stress (Cheng et al., 2018). Magnolol is used as an antioxidant to treat coliform enteritis in clinical practice (Deng et al., 2017). But the protective effect of magnolol on ALD is still unclear. So we established the model of ALD to investigate the role of magnolol in alcohol-induced liver damage and then explored the underlying mechanisms. The results showed that magnolol effectively protected the liver by reducing oxidative stress and inflammation in alcohol-induced liver damage.

AST and ALT are common markers for determining liver damage. Our result showed the levels of AST and ALT increased by alcohol were increased, but magnolol pretreatment could markedly reduce the content of AST and ALT. In addition, pathological changes such as the liver necrosis, inflammatory cell infiltration, and lipid droplets were improved in the liver of the mice pretreated with magnolol compared with the alcohol group. The above results showed that we successfully built the model of ALD, but the underlying mechanism of how magnolol protected the liver from alcohol damage was unclear.

Oxidative stress is an important pathogenic mechanism of ALD. CYP2E1 is one of the main enzymes in MEOS and plays a vital role in ethanol metabolism (Doody et al., 2017; Yuan et al., 2018). After excessive drinking or drinking a high concentration of alcohol, the activity of CYP2E1 is promoted and resulting in excessive reactive oxygen species (ROS) (Chen., 2014; Zeng et al., 2018). Then large amounts of reducing substances in the tissue including SOD and GSH-Px are consumed; alcohol-induced GSH-Px and SOD consumption are thought to aggravate oxidative damage in ALD mice model (Cederbaum, 2013; Zeng et al., 2018). The remaining lots of ROS cannot be inactivated, which also promotes the production of toxic lipid intermediates by fatty acids through abnormal decomposition reactions, causing oxidative stress and damage to the liver (Chen et al., 2014; Zeng et al., 2018). Besides, several reports indicated that iNOS and Cox-2, which are key mediators, play roles in the development of liver damage (Li et al., 2017). Our results showed that magnolol significantly increased the activities of SOD and GSH-Px in the liver, and it effectively reduced the expression of CYP2E1, iNOS, and Cox-2, which revealed that magnolol pretreatment could improve antioxidant capacity of the host to prevent ALD.

The PI3K/AKT signaling pathway can be activated by a variety of different cell stimuli and toxins and can be involved in the regulation of many cellular processes (Zhao et al., 2016). Oxidative stress produced by ROS can lead to down-regulation of PI3K and AKT (Pan et al., 2017). Western blotting analysis showed a decrease in the expression of PI3K and AKT in the model group compared to the blank group. However, magnolol activated the PI3K/AKT signaling pathway in a dose-dependent manner. Research reports that Nrf2 is a downstream target of PI3K/AKT (J. Zhang et al., 2017), and PI3K/AKT signaling pathway can regulate Nrf2/HO-1 transcription (Pan et al., 2017; J. Zhang et al., 2017). So we next tested the prevention of magnolol to ALD through Nrf2/HO-1 signaling pathway.

It is reported that Nrf2/ARE (antioxidant response element) is a newly discovered defensive transduction pathway against external oxidation and chemical stimulation (Li et al., 2018). Under physiological conditions, the binding of Nrf2 and Keap1 (Kelch-like ECH-associated protein-1) in the cytoplasm is in a state of easy degradation (Zhu et al., 2016; Li et al., 2018). When stimulated by internal and external free radicals and chemicals, the conformation of Keap1 changes or Nrf2 is phosphorylated directly (Jiang et al., 2017). The activated Nrf2 enters the nucleus and binds to the antioxidant element ARE to activate the expression of downstream antioxidant protease and HO-1 to resist the internal and external stimulation (Loboda et al., 2016; Ge et al., 2017). Then the potential mechanism that Nrf2/HO-1signaling pathway plays in the protection of magnolol to the liver from ALD was asked. It was revealed that alcohol decreased the expression of Nrf2, but the pretreatment of magnolol could reverse the decline of Nrf2. However, the expression level of HO-1 in the alcohol group was increased compared to the control group. The same situation in other’s studies was also found that ethanol can induce increased expression of HO-1. Up-regulation of HO-1 may be one of the most critical cytoprotective mechanisms in cellular stress (Gong et al., 2003). Since HO-1 is an inducible enzyme, HO-1 exhibits an increased reactivity during the early stages of acute ethanol stimulation. However, the consumption of HO-1 in this process leads to a decline in its level until ALD develops into alcoholic hepatitis (Liu et al., 2018). This may explain why the expression level of HO-1 in alcohol group is higher than that in the control group in this study. And with the increase of the concentration of magnolol, the expression of HO-1 gradually increased in a dose-dependent way, indicating that magnolol could enhance the protective ability against ALD by Nrf2/HO-1 signaling pathway. In addition, western blotting analysis showed that the protein expression of Nrf2 and HO-1 could be increased to normal level at low concentration of magnolol, while the protein expression of other signaling pathways could be the normal level only at medium or high concentration, suggesting that Nrf2 and HO-1 signaling pathway may be the key target of magnolol action.

PPARγ is a subtype of PPARs (peroxisome proliferator-activated receptors), which controls many intracellular metabolic processes and belongs to ligand-induced nuclear receptors (Rosen and Spiegelman, 2001). It is reported that Nrf2 regulates PPARγ, and the expression of PPARγ in Nrf2-knockout mice is significantly reduced under oxidative stress (Lee, 2017). It was also found that Nrf2 and PPARγ are mutually regulated, and the two pathways are positive feedback (Reddy and Standiford, 2010). PPARα, another member of the PPAR family, plays a key role in regulating liver fatty acid oxidation, and long-term drinking can cause a decrease in PPARα expression in the liver. Administration of PPARα agonists improves liver disease in mice caused by chronic alcohol exposure (Chen et al., 2017; Ding et al., 2017). Sterol regulatory element binding protein-1c (SREBP-1c) is an intracellular cholesterol sensor located in the endoplasmic reticulum. Overexpression of SREBP-1c increases ROS levels in hepatocytes and aggravates inflammatory damage in liver tissue, whereas activation of PPAR-α inhibits SREBP-1c signaling pathway (Ren et al., 2017). Our results showed that the expression of PPARγ in the alcohol group had decreased, but the expression in the magnolol pretreatment groups was increased. It revealed that magnolol could improve the antioxidant ability through activating PPARγ to prevent the liver from ALD.

In addition to oxidative stress, excessive inflammatory response in the liver is also another general disease mechanism recognized by ALD. Studies have shown that pro-inflammatory factors like IL-1β and TNF-α can aggravate the degree of liver damage (Sun et al., 2018). Our results revealed magnolol pretreatment effectively reduced the secretion of IL-1β and TNF-α in ALD. Besides, NLRP3 inflammasome plays a key role in many disease processes and it has the potential to bridge the link between inflammatory and oxidative stress responses (Haneklaus and O’Neill, 2015; Hughes and O’Neill, 2018). NLRP3 recruits and activates the pro-inflammatory protein Caspase-1, and activated Caspase-1 stimulates macrophage secretion of IL-1β to induce liver damage (Shao et al., 2015). Recent research reports that Nrf2, PI3K, and PPARγ can regulate the expression of NLRP3. Nrf2 prevents NLRP3 inflammasome activation by regulating Trx1/TXNIP complex in cerebral ischemia-reperfusion injury (Hou et al., 2018). PPARγ has an anti-inflammatory effect by inhibiting NLRP3 in spinal cord-derived neurons (Meng et al., 2019). Our results revealed that magnolol could down-regulate the activation of the NLRP3 inflammasome, caspase-1, and caspase-3 caused by alcohol, and then the downstream secretion of the pro-inflammatory cytokine IL-1β was inhibited, suggesting that magnolol has the ability to prevent liver from ALD by alleviating inflammatory response and oxidative stress.

In conclusion, our results confirmed that magnolol could prevent alcohol-induced liver damage by inhibiting oxidative stress and inflammation. And this study also provided a potential basis for future clinical ALD treatment and research.

## Data Availability Statement

All datasets generated for this study are included in the article/**Supplementary Material**.

## Ethics Statement

The animal study was reviewed and approved by Care and Use of Laboratory Animals of the Jilin University.

## Author Contributions

XL, YW, DW, SL, CW, ZH, and JW assisted in carrying out the experiment. XL wrote the manuscript. ZW, ZY, and KW helped with the design of experimental ideas and the revision of manuscripts.

## Funding

This work was funded by the National Natural Science Foundation of China (No. 31772721).

## Conflict of Interest

The authors declare that the research was conducted in the absence of any commercial or financial relationships that could be construed as a potential conflict of interest.

## Supplementary Material

Data Sheet 1 The raw data of Western Blotting analysis.

Data Sheet 2 The detection of iNOS, Cox-2, and CYP2E1 atthe enzyme level.
