# Supplementary figures and images for: Magnolol Prevents Acute Alcoholic Liver Damage by Activating PI3K/Nrf2/PPARγ and Inhibiting NLRP3 Signaling Pathway
Source: Front Pharmacol. 2019 Dec 5;10:1459. doi: 10.3389/fphar.2019.01459 (PMC6915046; doi:10.3389/fphar.2019.01459)

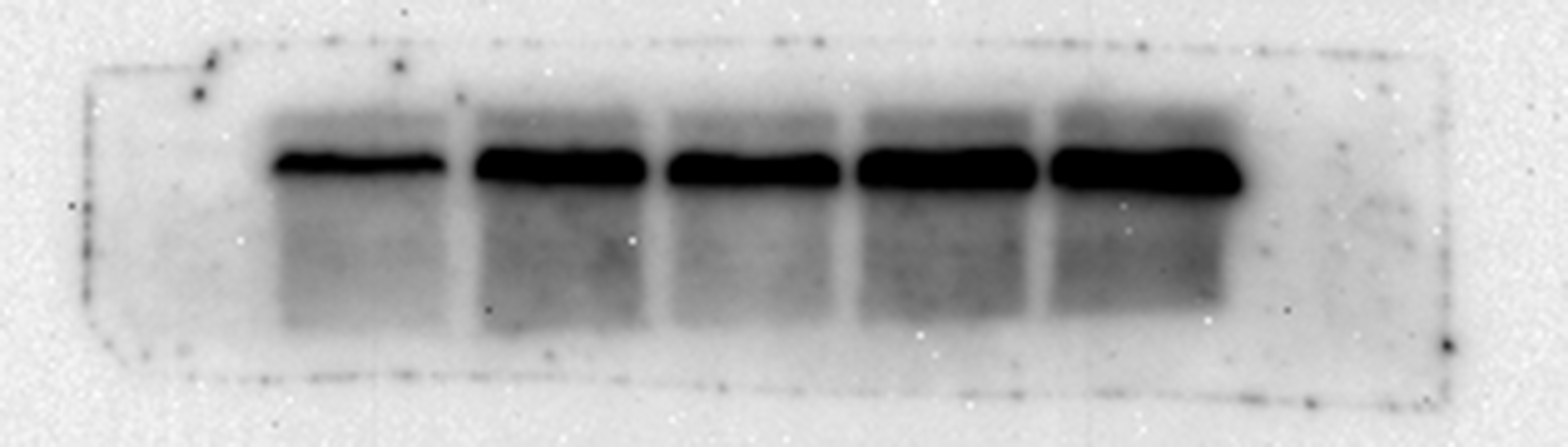

Supplement: Data Sheet 1 — The raw data of Western Blotting analysis. [file DataSheet_1.zip › WB analysis/AKT/1.tif]

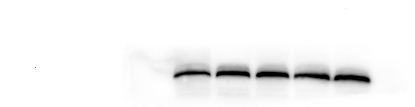

Supplement: Data Sheet 1 — The raw data of Western Blotting analysis. [file DataSheet_1.zip › WB analysis/AKT/2.png]

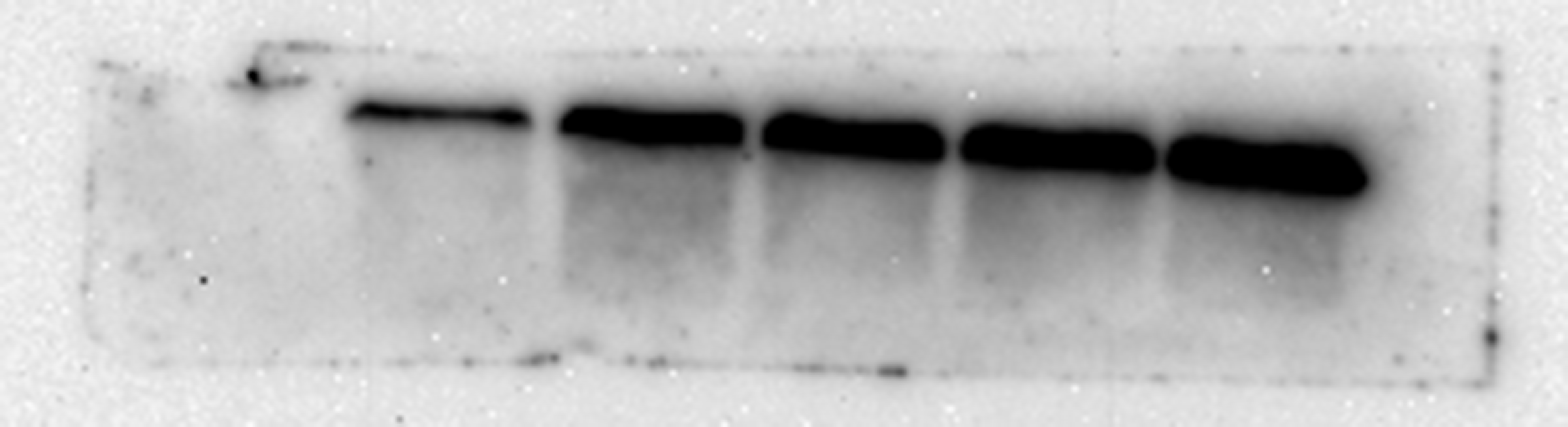

Supplement: Data Sheet 1 — The raw data of Western Blotting analysis. [file DataSheet_1.zip › WB analysis/AKT/3.tif]

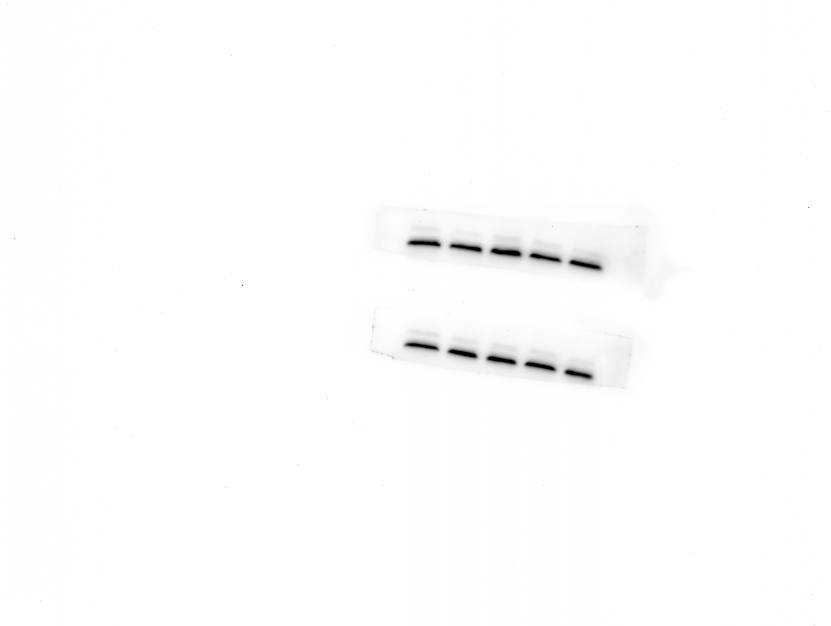

Supplement: Data Sheet 1 — The raw data of Western Blotting analysis. [file DataSheet_1.zip › WB analysis/GAPDH/1-2.png]

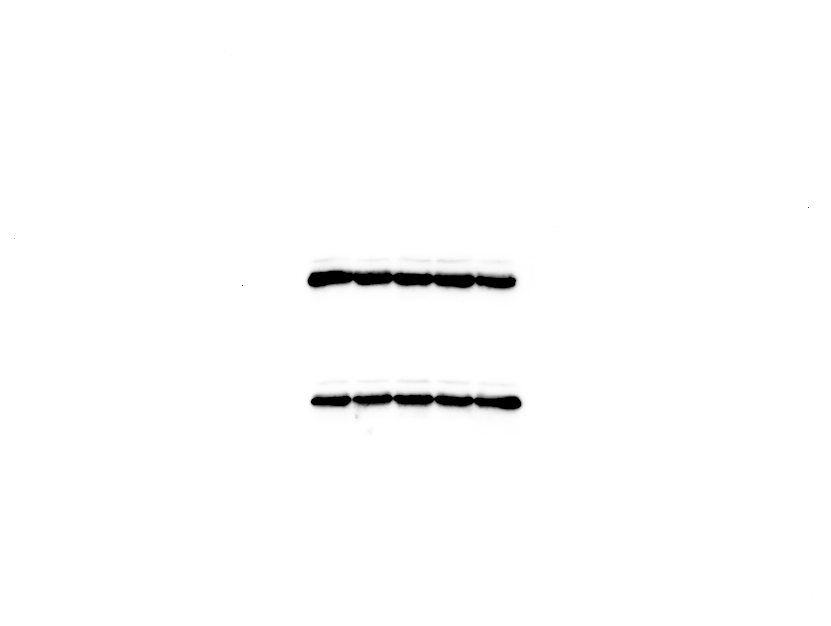

Supplement: Data Sheet 1 — The raw data of Western Blotting analysis. [file DataSheet_1.zip › WB analysis/GAPDH/3-4.png]

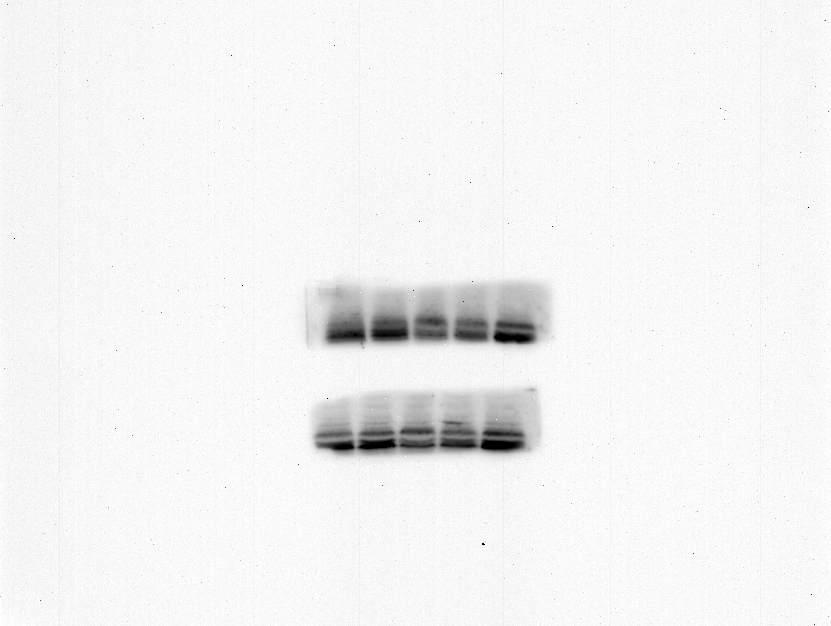

Supplement: Data Sheet 1 — The raw data of Western Blotting analysis. [file DataSheet_1.zip › WB analysis/HO-1/1-2.png]

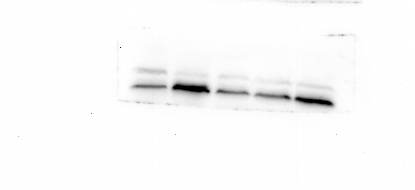

Supplement: Data Sheet 1 — The raw data of Western Blotting analysis. [file DataSheet_1.zip › WB analysis/HO-1/3.png]

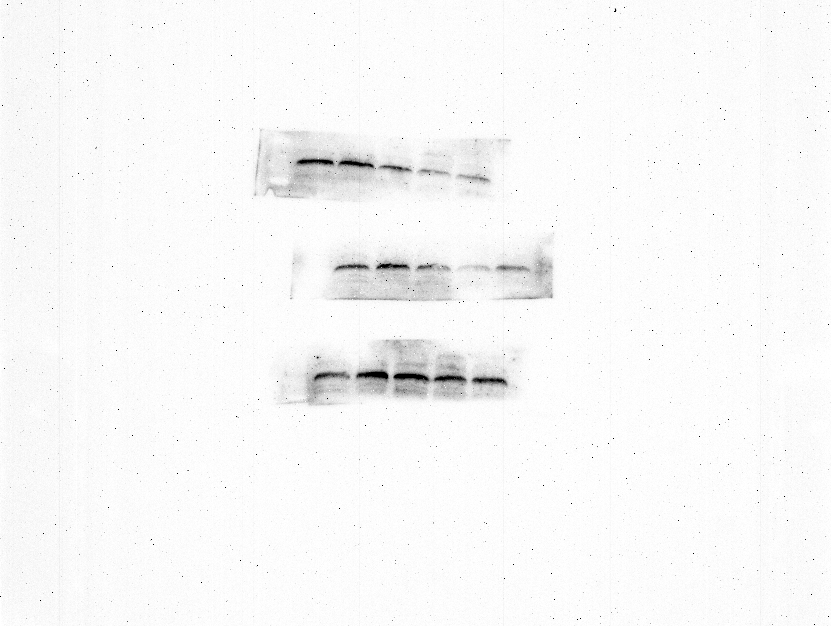

Supplement: Data Sheet 1 — The raw data of Western Blotting analysis. [file DataSheet_1.zip › WB analysis/NLRP3/1-2.png]

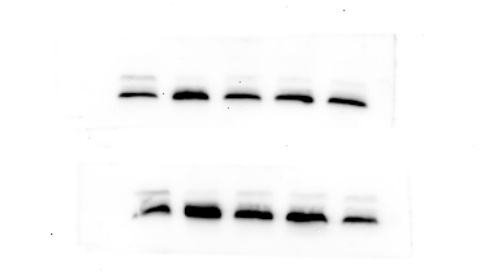

Supplement: Data Sheet 1 — The raw data of Western Blotting analysis. [file DataSheet_1.zip › WB analysis/NLRP3/3-4.png]

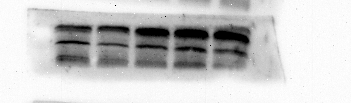

Supplement: Data Sheet 1 — The raw data of Western Blotting analysis. [file DataSheet_1.zip › WB analysis/Nrf2/1.png]

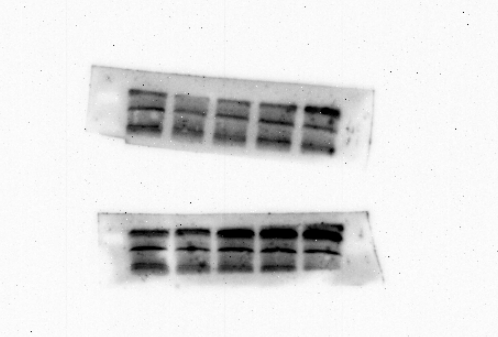

Supplement: Data Sheet 1 — The raw data of Western Blotting analysis. [file DataSheet_1.zip › WB analysis/Nrf2/2-3.png]

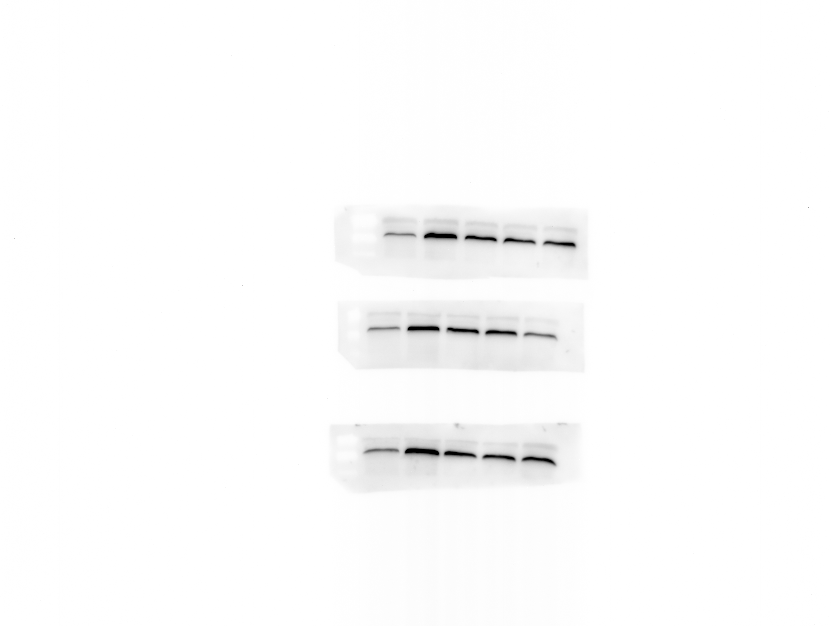

Supplement: Data Sheet 1 — The raw data of Western Blotting analysis. [file DataSheet_1.zip › WB analysis/PI3K/PI3K.png]

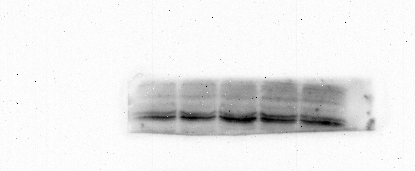

Supplement: Data Sheet 1 — The raw data of Western Blotting analysis. [file DataSheet_1.zip › WB analysis/PPARa├/1.png]

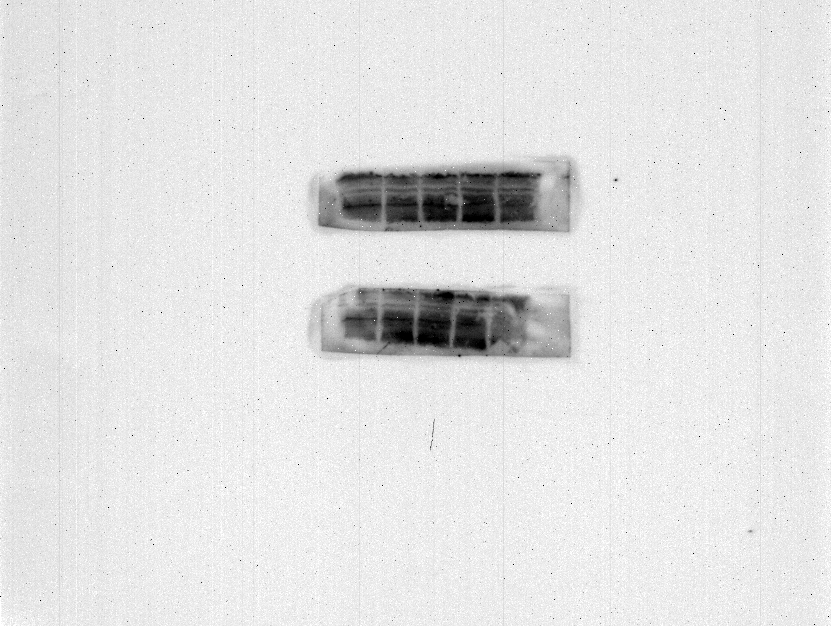

Supplement: Data Sheet 1 — The raw data of Western Blotting analysis. [file DataSheet_1.zip › WB analysis/PPARa├/2-3.png]

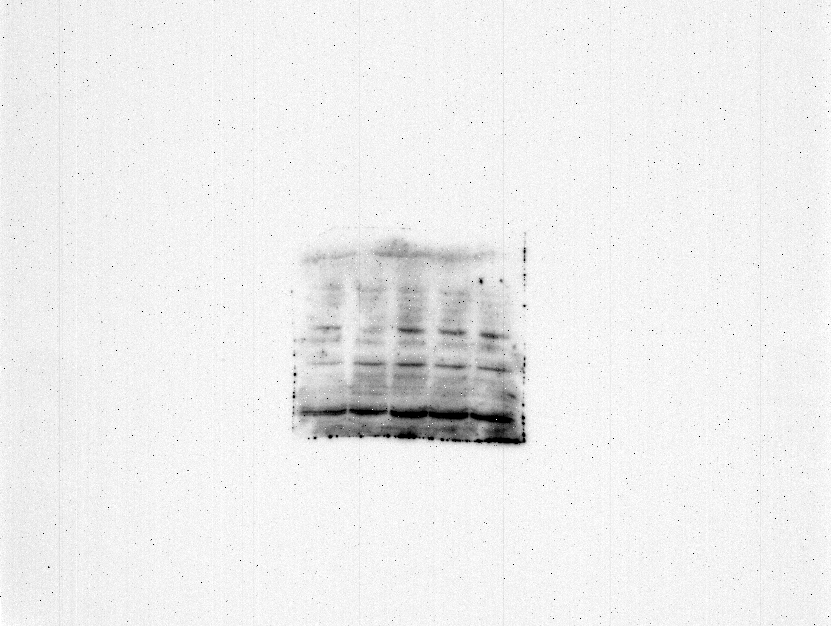

Supplement: Data Sheet 1 — The raw data of Western Blotting analysis. [file DataSheet_1.zip › WB analysis/caspase-1/1.png]

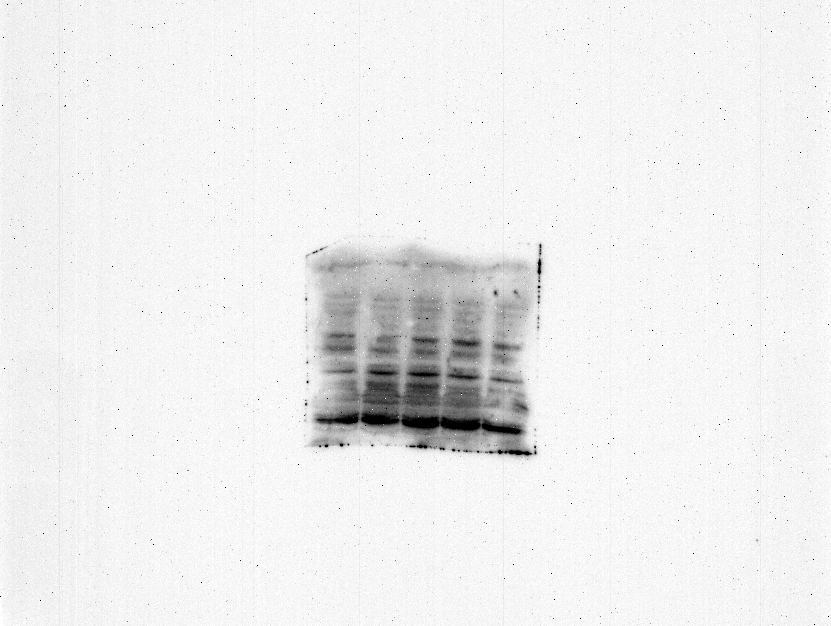

Supplement: Data Sheet 1 — The raw data of Western Blotting analysis. [file DataSheet_1.zip › WB analysis/caspase-1/2.png]

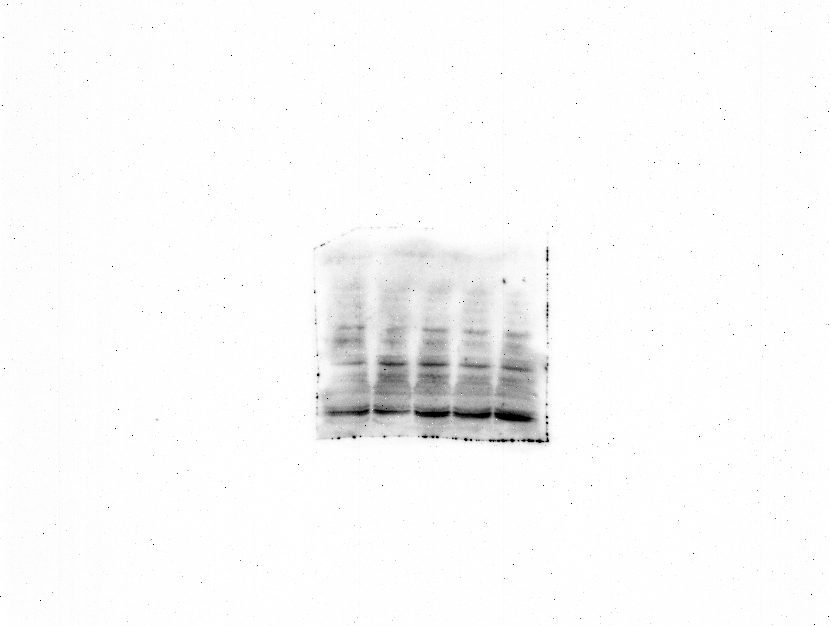

Supplement: Data Sheet 1 — The raw data of Western Blotting analysis. [file DataSheet_1.zip › WB analysis/caspase-1/3.png]

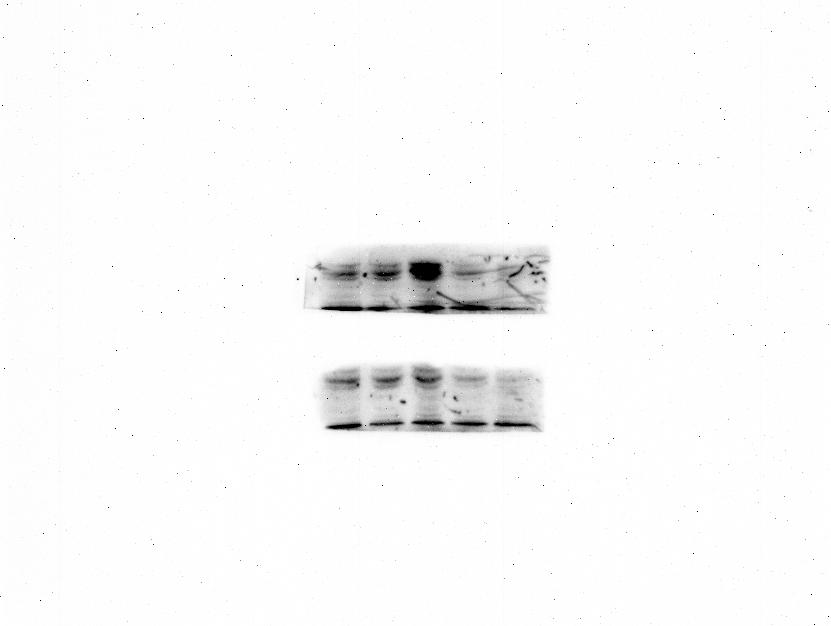

Supplement: Data Sheet 1 — The raw data of Western Blotting analysis. [file DataSheet_1.zip › WB analysis/caspase-3/1-2.png]

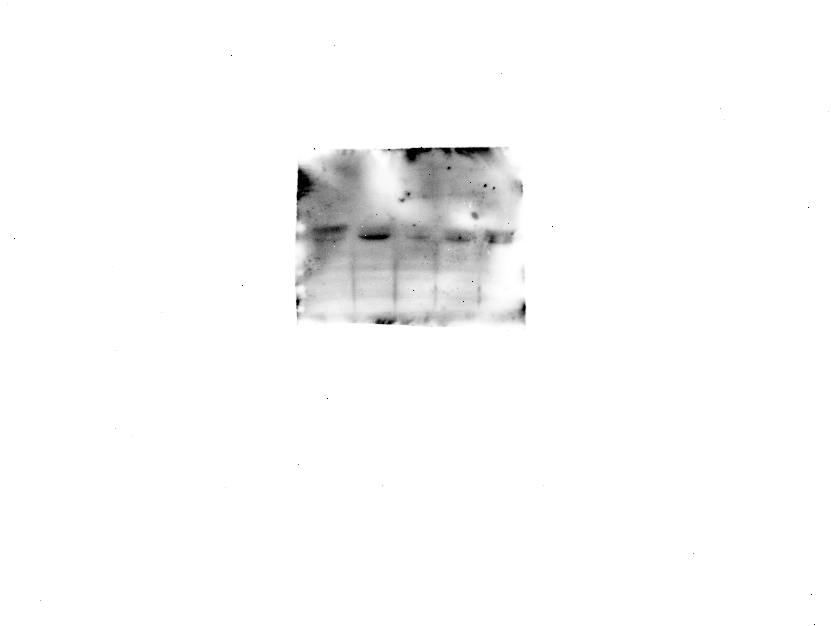

Supplement: Data Sheet 1 — The raw data of Western Blotting analysis. [file DataSheet_1.zip › WB analysis/caspase-3/3.png]

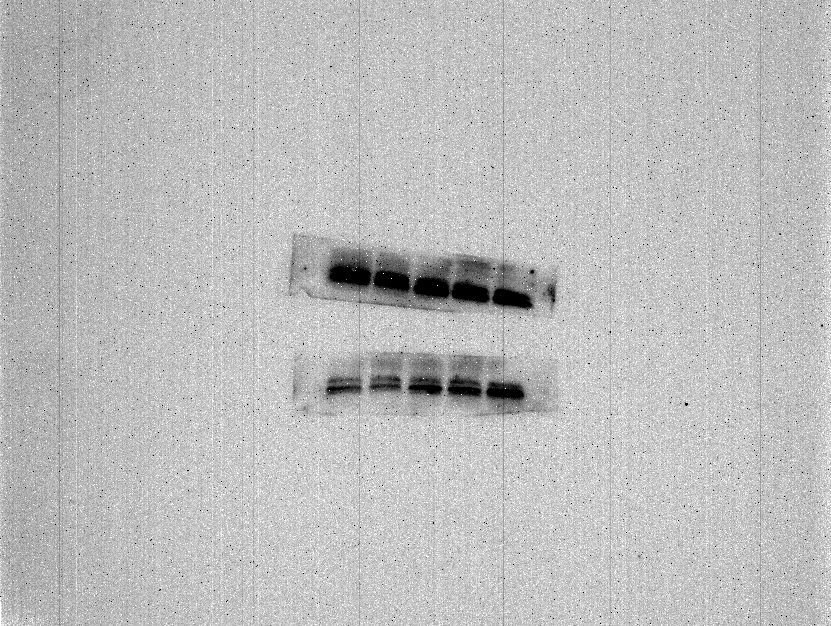

Supplement: Data Sheet 1 — The raw data of Western Blotting analysis. [file DataSheet_1.zip › WB analysis/p-AKT/1-2.png]

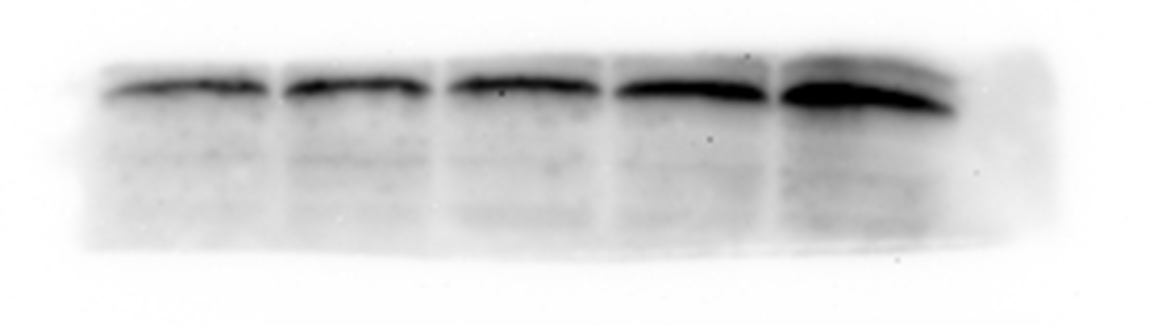

Supplement: Data Sheet 1 — The raw data of Western Blotting analysis. [file DataSheet_1.zip › WB analysis/p-AKT/3.tif]

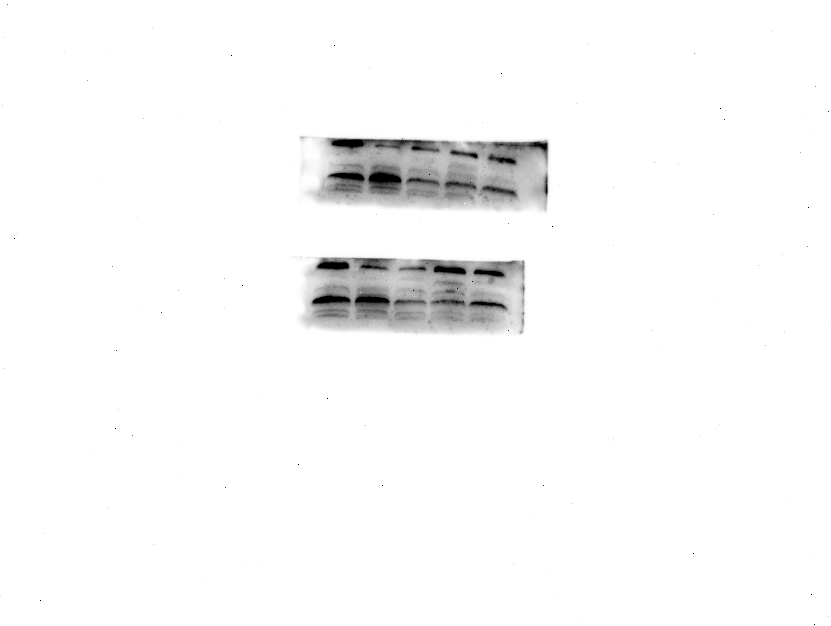

Supplement: Data Sheet 1 — The raw data of Western Blotting analysis. [file DataSheet_1.zip › WB analysis/p-PI3K/1-2.png]

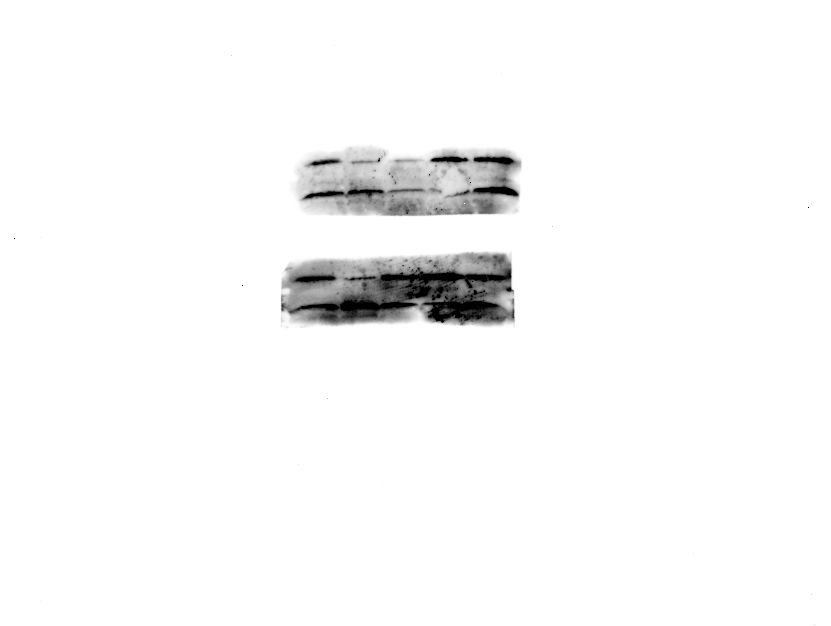

Supplement: Data Sheet 1 — The raw data of Western Blotting analysis. [file DataSheet_1.zip › WB analysis/p-PI3K/3-4.png]

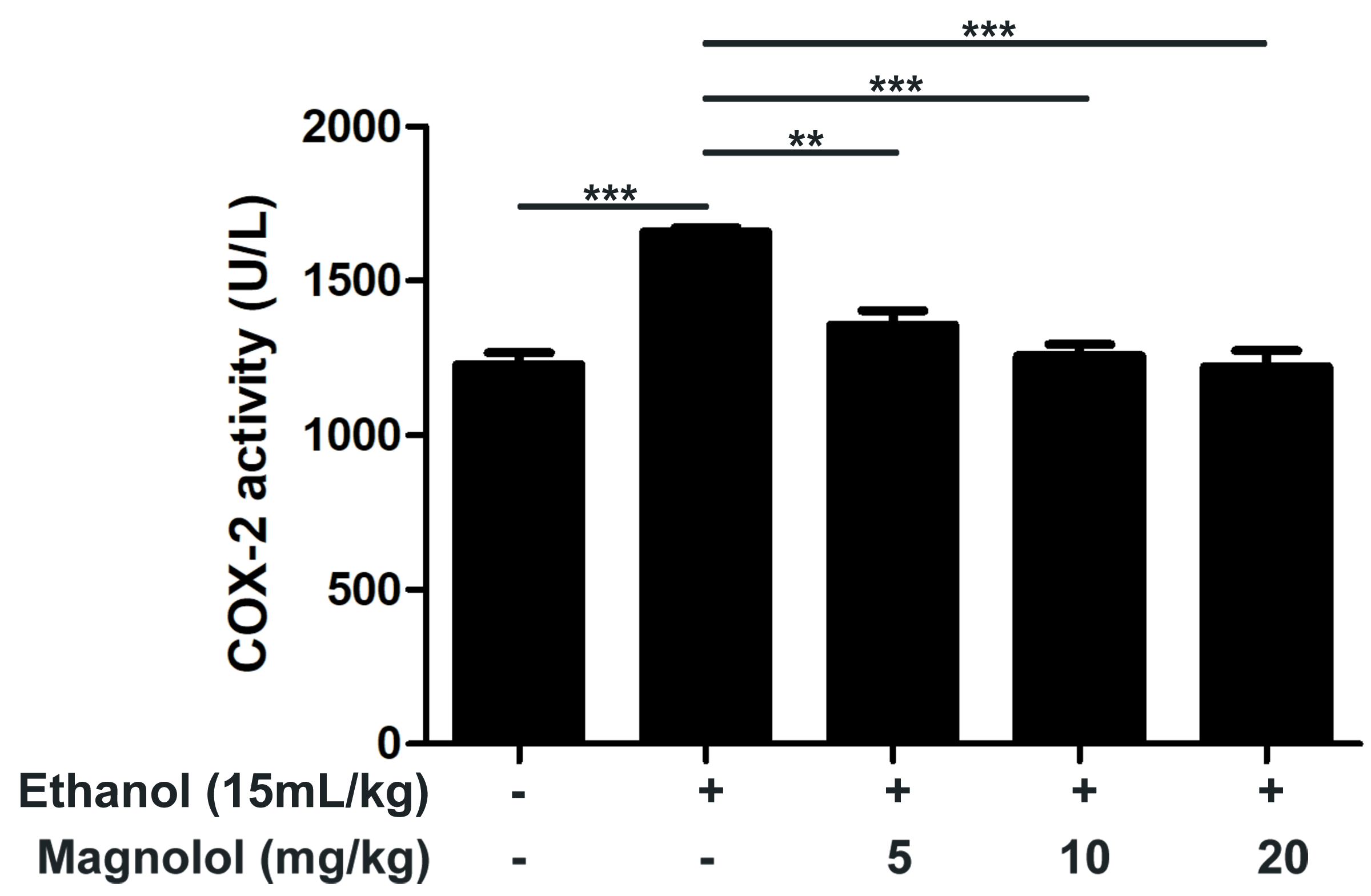

Supplement: Data Sheet 2 — The detection of iNOS, Cox-2, and CYP2E1 atthe enzyme level. [file DataSheet_2.zip › Supplementary material of COX-2, CYP2E1, iNOS/COX2/1.tif]

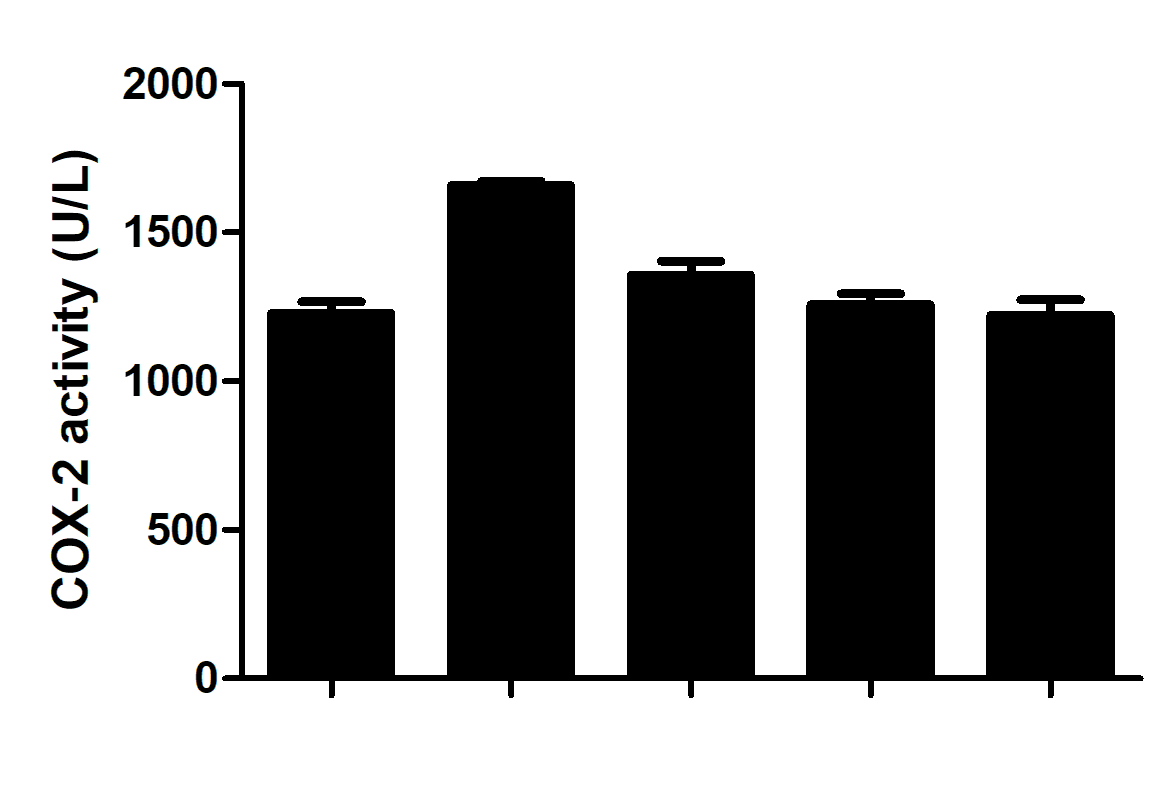

Supplement: Data Sheet 2 — The detection of iNOS, Cox-2, and CYP2E1 atthe enzyme level. [file DataSheet_2.zip › Supplementary material of COX-2, CYP2E1, iNOS/COX2/Data 1.tif]

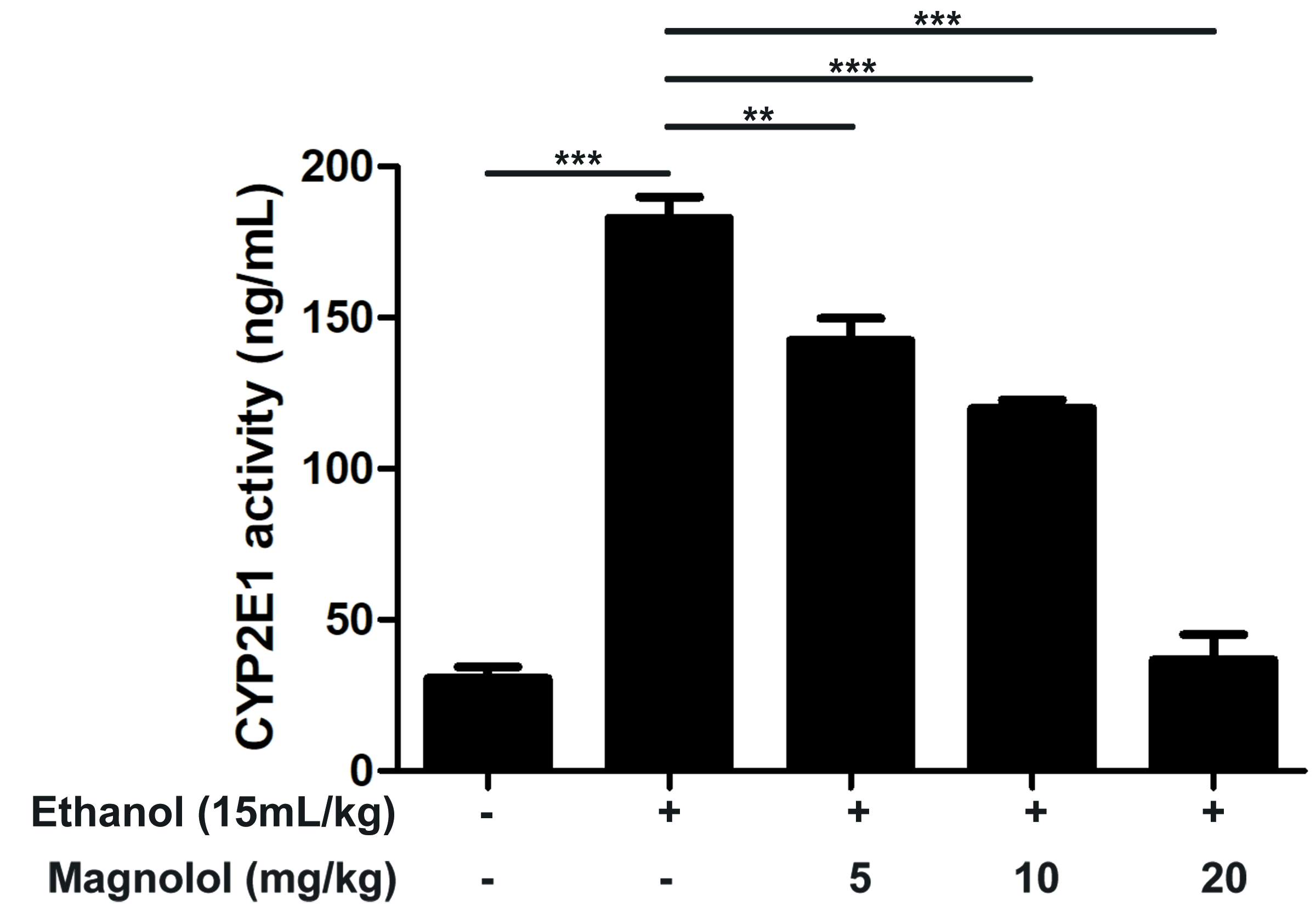

Supplement: Data Sheet 2 — The detection of iNOS, Cox-2, and CYP2E1 atthe enzyme level. [file DataSheet_2.zip › Supplementary material of COX-2, CYP2E1, iNOS/CYP2E1/1.tif]

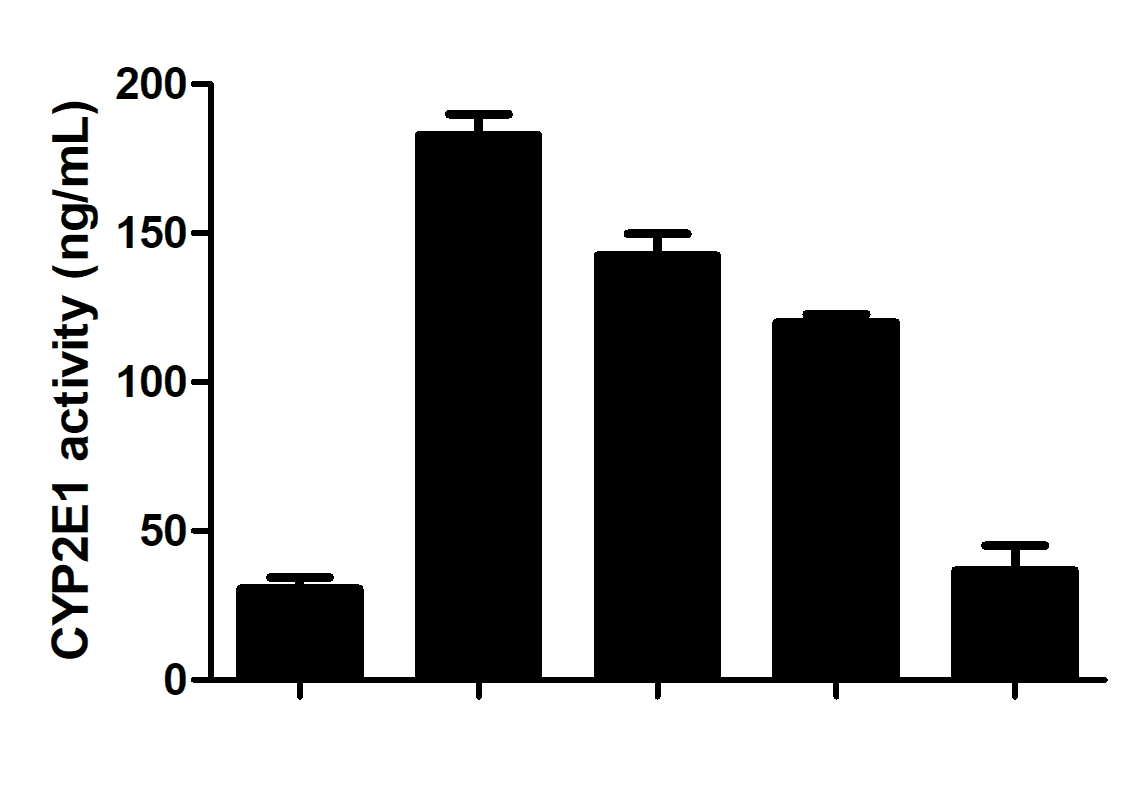

Supplement: Data Sheet 2 — The detection of iNOS, Cox-2, and CYP2E1 atthe enzyme level. [file DataSheet_2.zip › Supplementary material of COX-2, CYP2E1, iNOS/CYP2E1/Data 1.tif]

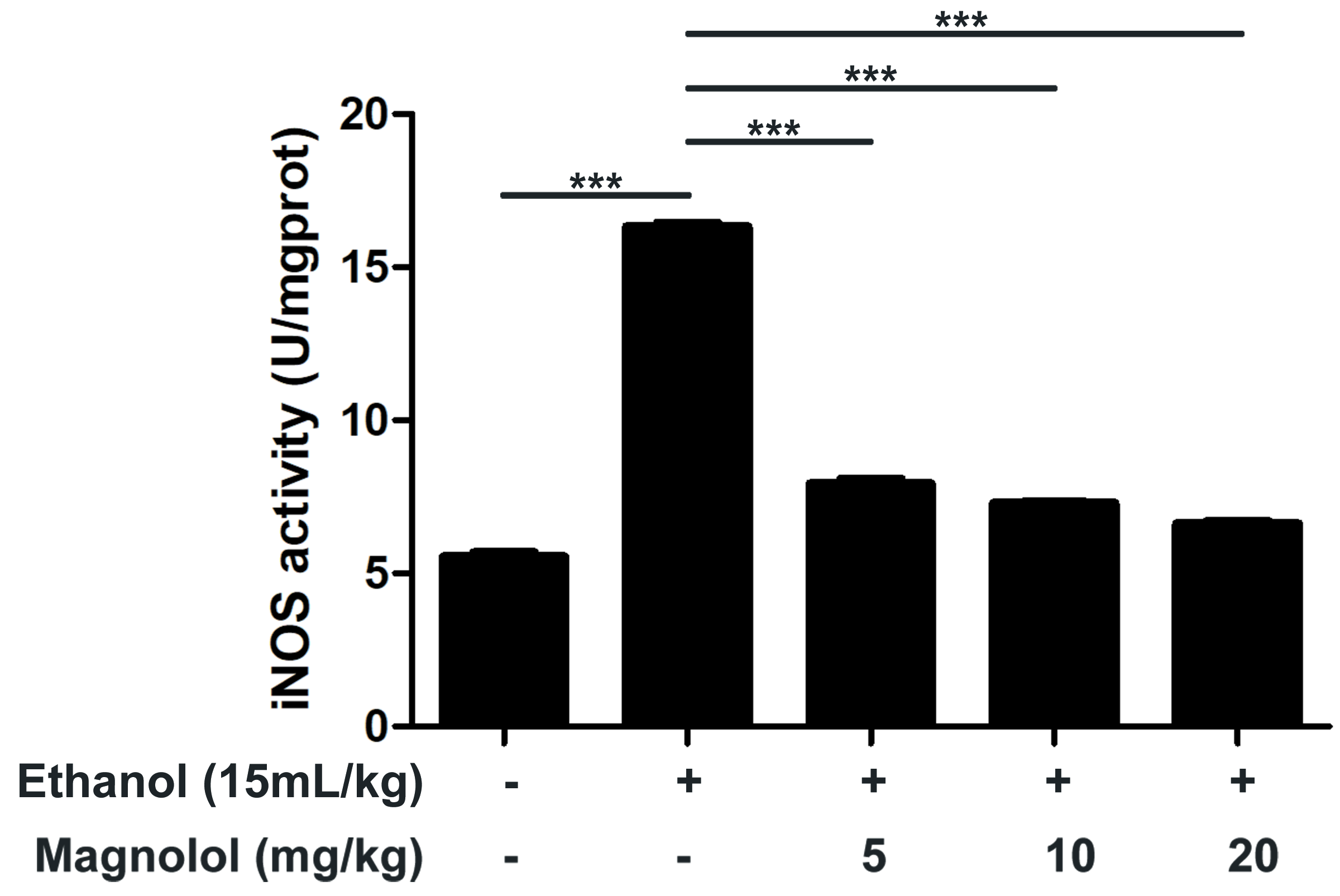

Supplement: Data Sheet 2 — The detection of iNOS, Cox-2, and CYP2E1 atthe enzyme level. [file DataSheet_2.zip › Supplementary material of COX-2, CYP2E1, iNOS/iNOS/1.tif]

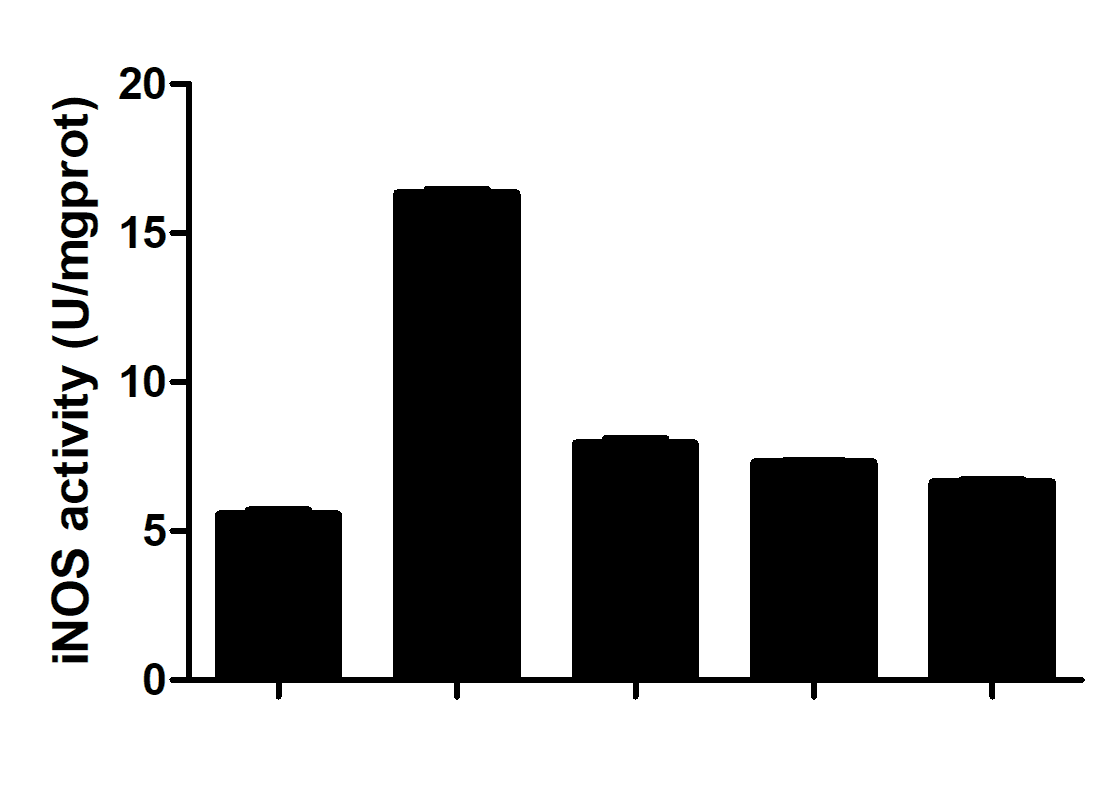

Supplement: Data Sheet 2 — The detection of iNOS, Cox-2, and CYP2E1 atthe enzyme level. [file DataSheet_2.zip › Supplementary material of COX-2, CYP2E1, iNOS/iNOS/Data 1.tif]
